# Arbuscular Mycorrhizal Fungi as Biostimulant and Biocontrol Agents: A Review

**DOI:** 10.3390/microorganisms12071281

**Published:** 2024-06-24

**Authors:** Mathieu Delaeter, Maryline Magnin-Robert, Béatrice Randoux, Anissa Lounès-Hadj Sahraoui

**Affiliations:** Unité de Chimie Environnementale et Interactions sur le Vivant (UCEIV, UR 4492), Université du Littoral Côte d’Opale, 50 Rue Ferdinand Buisson, 62228 Calais, France

**Keywords:** mycorrhizal inoculum, sustainable agriculture, biostimulation, plant protection

## Abstract

Arbuscular mycorrhizal fungi (AMF) are soil microorganisms living in symbiosis with most terrestrial plants. They are known to improve plant tolerance to numerous abiotic and biotic stresses through the systemic induction of resistance mechanisms. With the aim of developing more sustainable agriculture, reducing the use of chemical inputs is becoming a major concern. After providing an overview on AMF history, phylogeny, development cycle and symbiosis benefits, the current review aims to explore the potential of AMF as biostimulants and/or biocontrol agents. Nowadays, AMF inoculums are already increasingly used as biostimulants, improving mineral nutrient plant acquisition. However, their role as a promising tool in the biocontrol market, as an alternative to chemical phytosanitary products, is underexplored and underdiscussed. Thus, in the current review, we will address the mechanisms of mycorrhized plant resistance to biotic stresses induced by AMF, and highlight the various factors in favor of inoculum application, but also the challenges that remain to be overcome.

## 1. Introduction

Conventional agronomic techniques, such as agrochemical inputs, have made it possible to guarantee access to food for humanity [1]. Nowadays, the human population keeps growing, approaching 9.7 billion people in 2050 according to the United Nations [2]. However, conventional agriculture is showing its limitations, raising social concerns and is usually decried for its impact on all ecosystems, both on wildlife and human society (pollution, toxicity, reduced effectiveness of chemical plant protection products, destruction of ecological niches for the benefit of invasive species, etc.) [1,3,4,5,6,7]. Numerous policies and agronomic practices are being implemented around the world to foster a reduction in the use of chemical inputs, like plant protection products. For instance, in 2022, the European Commission adopted the proposal 2022/0196 for the regulation (EU) 2021/2115, which aims to reduce at least 50% of chemical plant protection products by 2030 [8]. Integrated pest management (IPM) implies the use of agronomic practices to stop or limit diseases and pest growth in the field, using as few as possible chemical phytosanitary products and preferably employing sustainable techniques and products [9]. The practice of organic farming prohibits the use of synthetic inputs and chemical phytosanitary products, as set out in the European directive 2018/848 [10]. Within this context, the agricultural world really needs alternatives to the synthetic products used in conventional farming.

Currently, biostimulant products, defined by du Jardin [11] as “any substance or microorganism applied to plants to improve nutritional efficiency, tolerance to abiotic stresses and/or important quality characteristics of the crop, irrespective of its nutrient content”, are playing an important role in the agricultural products’ market for substituting or limiting chemical fertilizers. Among them, beneficial microorganisms are increasingly used as biostimulant products, notably in fields, horticulture and forestry [12]. These microorganisms are well-known as “Plant Growth Promoting Rhizobacteria” (PGPR) (e.g., *Azotobacter* sp., *Rhizobium* sp., *Azospirillum* sp.) [13] and “Plant Growth-Promoting Fungi” (PGPF) (e.g., *Trichoderma* sp.) [14,15], which act as a support to crop growth, enhancers of nutrient supplies to plants (phosphate, nitrogen, etc.) and stimulants of plant organ development [13,16].

For many years, Glomeromycetes, a specific lineage of fungi and commonly called “arbuscular mycorrhizal fungi” (AMF) have drawn the attention of the scientific community. Like PGPR and PGPF, AMF are already used for their ability to improve plant nutrition and growth [17], and have been classified in the European Union as biostimulant products according to the recent EU regulation 2019/1009 [18]. Also, they are often studied as interesting tools against diverse agricultural issues strengthened by climate change and mainly due to pedoclimatic stresses such as hydric, thermal, saline, osmotic, luminous, pollution and nutritional stresses [19].

Alongside their biostimulant abilities, AMF have also been studied in numerous biotic stress conditions. It has been shown that AMF are able to induce defense mechanisms leading to a “Mycorrhiza-Induced Resistance” (MIR) [20], resulting in protection against attacks by various pests. For decades, controlling pest and crop diseases in conventional agriculture has mainly focused on the application of chemical pesticides and plant breeding. As an alternative, biocontrol products, which limit pest and disease development in a more sustainable way by copying or using direct, natural mechanisms [21], already employ a wide range of beneficial microorganisms such as PGPR and PGPF [22]. Thanks to their abilities to induce the plants’ defenses, AMF could be used as biocontrol agents.

Firstly, this review aims to provide an overview of the history, phylogeny and development of AMF and plant symbiosis. Subsequently, the review will address their abilities to stimulate plant nutrition and growth, making them ideal for use as biostimulant products to replace chemical inputs, and to be recognized as plant tolerance inducers in front of numerous abiotic stresses. AMF could also take their place in the biocontrol industry, which is an eco-responsible alternative market to synthetic phytosanitary products used in conventional agriculture. The current state of research shows that this AMF potential in biocontrol has its pros and cons. Thus, the present review aims to examine the potential of AMF as plant protection agents, by exploring the plant defense mechanisms induced by mycorrhization upstream and during attacks by crop pests and diseases. Then, the review will state the persistent limitations of AMF for crop protection, which require further research to confirm their biocontrol potential. Finally, a brief review of AMF inoculum production will be carried out to examine whether current agribusinesses can extend the use of AMF inoculum to the biocontrol market.

## 2. Arbuscular Mycorrhizal Fungi (AMF)

### 2.1. History and Taxonomy

At the root level of most terrestrial plants, many edaphic fungi, described as “mycorrhizal” by Frank (1885) [23], form a symbiotic relationship with plants. These include a monophyletic line of endophytic fungi known as “arbuscular mycorrhizal fungi”. They were described firstly by von Nägeli in 1842 [24]. AMF are unable to mineralize soil organic matter due to their poor exo-enzymatic equipment [25]. Thus, to complete their development cycle, they have to establish compatible mutualistic symbiosis with host plants [26]. AMF are therefore obligate biotrophs [27]. AMF form a highly specific interface with the roots of the majority of vascular plants (spermatophytes, monilophyta) [28], except for certain families of *Chenopodiaceae*, *Brassicaceae* and *Proteaceae*, and certain genera of *Cactaceae* and *Fabaceae* families [29]. AMF have co-evolved with these plants since their appearance in the Ordovician period, 480 million years ago [30].

In the past, AMF were identified by studying the morphological structures of the spores. However, this method often led to inability or the misclassification of AMF because it is difficult to point out the morphological differences using microscope observation [31]. Nowadays, AMF taxonomy is based on the sequencing of certain parts of the AMF genomes, using numerous molecular markers such as β-tubulin gene [32], nuclear large subunit 28S (LSU) rRNA gene [33], nuclear small subunit 18S (SSU) rRNA gene [34], the internal transcribed spacer (ITS) comprising the 5.8 rRNA gene [35]. Currently, the AMF group belongs to the Glomeromycota phylum and the Mucoromyceta sub-range of fungi [36].

According to the AMF Phylogeny in 2024 (http://www.amf-phylogeny.com/, accessed on 20 May 2024), at least 352 species have been recorded to date. The taxonomic classification of AMF is evolving regularly. According to the last AMF classification proposed by Wijayawardene et al. [37], Glomeromycetes are subdivided into three classes (Archaeosporomycetes, Glomeromycetes, Paraglomeromycetes) and five orders that are Paraglomerales, Archaeosporales, Entrophosporales [38], Glomerales and Diversisporales [39,40] (Table 1).

### 2.2. Developmental Cycle

Mycorrhizal symbiosis establishment begins with a pre-symbiotic phase. In an unfavorable environment, particularly in low inorganic phosphate soils, the roots secrete exudates into the soil (branching factors), like strigolactones, enabling the mycorrhizal symbiosis to be established [56,57]. Observed for the first time in the symbiosis between *Lotus japonicus* and *Gigaspora margarita* [58], strigolactones induce AMF spore germination [59]. The AMF metabolism is changed, such as respiration, mitochondrial reorganization and lipid catabolism [60], which act on the growth and branching of AMF extra-radical hyphae, leading to the formation of a new primary mycelium [61]. Without signals from plants, AMF stops growing, apical hyphae vacuolize, hyphae are compartmentalized by septa and a retraction of cell cytoplasm and nuclei happens [62,63]. Then, the AMF itself emits molecules towards the roots called “Myc factors” [64], such as lipochitooligosaccharides (LCOs) which activate the genes involved in symbiosis establishment in the plant roots [65].

**Figure 1:**
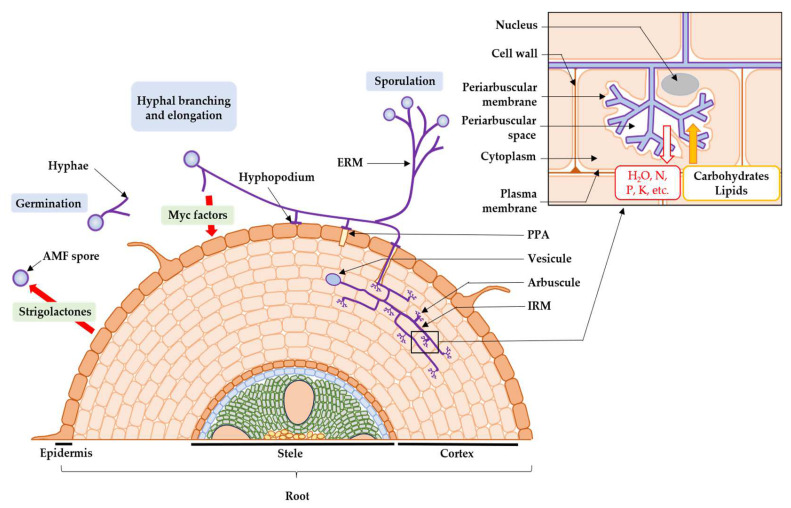
Developmental cycle of AMF (ERM: extra-radical mycelium; IRM: intra-radical mycelium; PPA: prepenetration apparatus).

Then, the symbiotic phase of the fungus begins (Figure 1). On the arrival of AMF, the cells of the root epidermis modify their structures to form a “prepenetration apparatus” (PPA), which determines the entry of AMF into the root [66,67]. Then, the AMF forms a hyphopodium that burrows into the epidermal cell layer, with intra-radical mycelium colonizing the extracellular space between the cortical cells [66,67]. Intra-radical hyphae penetrate these cells and form arbuscules [68], branched structures where the bidirectional exchange of signal molecules and nutrients occurs between the two partners in the symbiosis [66,67]. Theses arbuscules and cells are separated by the periarbuscular space [66,67]. The plant exchanges up to 20% of its carbon molecules from photosynthesis with AMF [69,70], in the form of carbohydrates [71] and fatty acids [72], against various macro- and micro-nutrients, taken from the soil via the extra-radical mycelium [73], such as phosphates [74], sulphates [75], nitrogen molecules [76], potassium [77], copper [77], iron [73,78], zinc [78], calcium, manganese, magnesium [79], and finally water [80,81]. Arbuscules have an average lifespan of 8.5 days, during which they are active for up to 5 days [82], and disappear by senescence, leaving the plant cell in its original state [83]. Intra-radical hyphae can also form other structures such as coiled hyphae and vesicles. These circular storage organs allow AMF to accumulate carbon in lipids for their own growth when photosynthesis does not produce sufficient carbon compounds [84]. Finally, new AMF spores are produced from extra-radical hyphae [85].

## 3. AMF as Biostimulant Agents

### 3.1. Plant Growth and Yield Improvements

**Figure 2:**
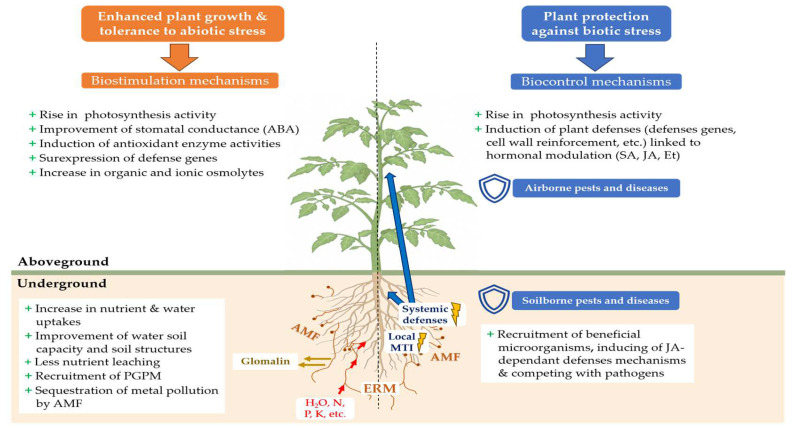
Main mechanisms of tolerance to biotic and abiotic stresses induced in the host plant after inoculation with AMF (ABA: abscisic acid; Et: ethylene; ERM: extra-radical mycelium; JA: jasmonic acid; MTI: MAMP-triggered Immunity; SA: salicylic acid; PGPM: plant growth-promoting microorganisms).

AMF provide naturally various benefits to their host plants. Indirectly, AMF improve soil characteristics which are beneficial for plant growth (Figure 2). AMF increase soil resistance to water and wind erosion [86] by improving soil structure thanks to the excretion of glomalin, a glycoprotein acting as a biological glue binding soil particles together to form larger soil aggregates [87,88]. AMF can enhance soil water holding capacity [89] and reduce nutrient leaching, particularly those crucial for plant cultivation such as nitrogen and phosphate [90]. Also, AMF naturally contribute to better carbon sequestration in soils [89,91]: the improvement of soil aggregation by glomalin prevents the degradation and the leaching of soil organic matter [92].

AMF absorb carbon molecules from the plant’s photosynthesis for their development and functioning [69,70]. This is not without consequences for the plant, because it creates a carbon sink for the plant: AMF will modify the primary carbon metabolism of the mycorrhizal plant, by deregulating photosynthesis and reducing the accumulation of photosynthates, to such an extent that a slowdown in growth has sometimes been observed in the establishment stage of AMF–plant symbiosis [93,94]. Under these conditions, the plant’s photosynthesis rate increases to compensate for carbon loss [95,96]. After mitigating carbon loss, these increases in photosynthesis, added to enhance nutrient supply, can stimulate plant development and growth (Figure 2). In practical terms, this means that mycorrhizal inoculation can directly improve yield and crop qualities. Field trials using mycorrhizal inoculation have demonstrated a significant increase in yields for potatoes [97], maize [98] and yams [99]. Gao et al. [100] reported that cotton plants (*Gossypium hirsutum*) inoculated in the field with *Rhizophagus irregularis* CD1 produced higher yields of cotton, with more cotton bowl and higher quality cotton fibers, thanks to an increase in phosphate concentration in the plants. Sorghum plants (*Sorghum bicolor*) growing in phosphate-poor soils and inoculated with *R. irregularis* produced higher yields and grains with superior nutrition, richer in phosphate, iron and zinc [78]. Zhang et al. [101] showed that, in mycorrhized rice plants, more nitrogen and carbon were observed in all the organs of the rice plants, and this had a positive influence on yield (up 28.2%) and protein concentration (up 7.4%) compared with non-inoculated plants. The composition in flavonoids, sugars, vitamin C, organic acids and minerals in the fruit of lemon trees mycorrhized by *Glomus versiforme* can be enriched [102]. Anthocyanins, carotenoids and other phenolic compounds are increased in lettuce in response to *Rhizophagus fasciculatus*, *R. irregularis* and *Funneliformis mosseae* inoculations [103]. AMF can also boost essential oil yields in medicinal and aromatic plants and improve their composition in terpenoids and flavonoids [104,105,106]. For all these reasons, mycorrhizal inoculation is becoming an agronomic tool among biofertilizers and plant biostimulants [107,108,109,110].

### 3.2. Plant Tolerance to Abiotic Stress

Widely documented in the scientific literature, biostimulation with AMF improves plant tolerance to numerous abiotic stresses. Through a meta-analysis of 546 publications between 1950 and 2021, Wu et al. [111] explained that, in countries where agriculture is rainwater-dependent and crops are regularly subjected to abiotic stresses such as drought or heat stress, mycorrhizal inoculations helped to increase harvested crop biomass by an average of 23%, especially in nitrogen-fixing plants, and average root biomass by up to 29.6% compared to non-mycorrhized cultivations. Under water and salt stress, the biomass of AMF-inoculated plants was increased [112,113,114]. Thanks to AMF, the plant tolerance to climatic hazards can provide food and economic security to human populations, and AMF inoculation becomes an interesting means to alleviate the deleterious effects of climatic change [115]. Symbiosis between AMF and the host plant improves the plant’ physiology, enabling them to cope with abiotic stresses, such as water, thermal (cold, heat), salt, pollution, osmotic and oxidative stresses [116,117,118,119,120,121]. This tolerance shown by AMF-inoculated plants is due to different mechanisms and changes occurring outside and inside the plants (Figure 2).

Mycorrhizal symbiosis improves plant mineral nutrition making the host plant more tolerant to abiotic stresses. Under salinity or metallic stresses, AMF can enhance plant mineral uptake, such as phosphate, nitrogen, iron, magnesium, copper or zinc [122,123,124]. Some mycorrhized plants have also been found to have higher concentrations of organic acids, which are used by plants to solubilize soil phosphate [125] and by AMF to mobilize essential mineral nutrients, such as N and P, that are difficult for plants to access [126]. Higher levels of organic acids enable mycorrhized plants to better tolerate alkaline [127] and saline [128] stresses.

AMF improve the plant’s water use under water, salt and cold stresses [116,119,129]. The hyphae of extra-radical mycelium, 2 to 5 µm thin, act as an extension of the plant’s roots, giving them access to micro-porosities in the soil that are difficult for the root’s absorbing hairs to reach, and to more distant regions of the soil for the root system [89,130,131]. Then, AMF induce the overexpression of aquaporin genes in the plant roots. Aquaporins (AQPs) are membrane transport proteins involved in water movement; the water transport is achieved via an osmotic gradient [132]. These aquaporins are, for example, PIP (plasma membrane intrinsic protein) 1 and 2, TIP (tonoplast intrinsic proteins) 1, 2 and 4, NIP (nodulin 26-like intrinsic protein) 1 and 2, SIP2 (small and basic intrinsic proteins 2), AQP2, and they all have been found to be involved in abiotic stresses such as drought stress [133,134,135,136,137,138], salinity stress [139] and cold stress [140]. In the roots of inoculated plants, aquaporin gene overexpression is beneficial for obtaining more water from the extra-radical mycelium, under low water availability [139]. In the above-ground organs of inoculated plants, the closure of stomata is induced by water or salt stresses, reducing the entry of CO_2_ into the plant and therefore photosynthesis activity [141]. In other cases, mycorrhizal symbiosis can moderate the consequences of these stresses, by inducing an increase in stomatal conductance, gas exchange, leaf water potential and photosynthesis, in particular chlorophyll levels [142]. In the face of low temperatures, mycorrhizal symbiosis can also reduce water loss [116] and an increase in leaf chlorophyll content [143]. Under water and salt stresses, the leaf area index is increased [113]. Finally, it should not be forgotten that glomalin, excreted by AMF, plays a positive role in soil structuring and facilitates water retention in the soil, this inevitably improves water availability for plants under abiotic stresses [86,87,88,89].

Linked to the enhancement of photosynthesis, a higher concentration of hexose is then observed in the tissues of the mycorrhized plants, which is partly metabolized by AMF into glucose [71] at the beginning of mycorrhizal symbiosis establishment [144] or in phosphorus-deficient conditions [70]. These higher sugar concentrations can be used by the mycorrhized plant for its own development [145]. Also, higher sugar concentrations give mycorrhized plants greater tolerance to various abiotic stresses such as drought [146], cold [147], high soil salinity [128,148] or pollution [149], because sugars can prevent structural changes in soluble proteins, maintain cell membrane integrity and osmotic balance.

In response to water, heat and salt stresses, the plant produces in excess “Reactive Oxygen Species” (ROS) such as hydrogen peroxide H_2_O_2_, superoxide anion radicals O_2_^−^, singlet oxygen ^1^O_2_ and hydroxyl radicals OH•, which can degrade by oxidation the plant’s nucleic, lipid, pigment and protein molecules [150,151,152,153]. This oxidative stress is countered by producing non-enzymatic antioxidant molecules (ascorbic acid, reduced glutathione, tocopherols, carotenoids, flavonoids, phenols, proline) [154,155,156,157]. It is also countered by enhancing antioxidant enzymatic activities (ascorbate peroxidase [APX], catalase [CAT], glutathione peroxidase [GPX], glutathione reductase [GR], peroxidases [POD], superoxide dismutase [SOD]) [154,155,156,157]. These antioxidant enzymes detoxify the ROS excess and maintain the cells’ homeostasis [154,155,156,157]. The stimulation of antioxidant enzyme activities has also been observed in the mycorrhized plant [158]. For example, Li et al. [159] attributed the preservation of the photosynthetic process in C_3_ and C_4_ plants through the stimulation of antioxidant enzyme activities in drought stress to mycorrhizal inoculation. During a salt stress affecting bread wheat plants (*Triticum aestivum* L.), Talaat and Shawky [160] observed that the leaves of wheat plants inoculated with *Glomus* spp. spores contained less H_2_O_2_ than those of non-inoculated plants.

Hormonal changes induced by mycorrhization, particularly the stimulation of abscisic acid (ABA) production, activate mechanisms for tolerance to water and salt stresses [161,162]. ABA prevents water loss [163] and osmotic and oxidative shocks by regulating the opening of stomatal guard cells (regulation of stomatal conductance) [164] and the up-regulation of defense genes against abiotic stresses such as SOD encoding genes [165]. Under metallic stresses, proline biosynthesis, root biomass or root nodulation and nutrient uptakes (iron, magnesium, phosphate, nitrogen) are stimulated by AMF in pigeon pea (*Cajanus cajan* (L.) Millsp.) [166].

Mycorrhizal symbiosis regulates osmotic adjustment in the tissues of inoculated plants stressed by lack of water, osmotic stress or excess salt. Because plants need to keep cellular processes and turgor pressure active, mycorrhized plants can accumulate organic osmolytes (glycine betaine, polyamines, prolines, soluble sugars) and ionic osmolytes (Ca^2+^, K^+^, Mg^2+^) [165,167,168,169].

Finally, AMF can detain the excess soil metallic ions in their hyphae, stopping them spreading into plant roots [121] and avoiding heavy metal pollution stress effects, such as the denaturation or modification of proteins and cell membrane disruptions [170]. Furthermore, it was observed that mycorrhizae induced metal fixation into their cell walls, accumulation into their vacuoles and chelation via siderophore into the cell cytoplasm [121]. As a result, mycorrhized plants cultivated under heavy metal stress developed better than non-mycorrhized plants [171].

## 4. AMF as Biocontrol Agents

According to the International Biocontrol Manufacturers’ Association (IBMA) [21], biocontrol products and agents offer sustainable pest and disease control strategies by using natural mechanisms. They include macro-organisms, chemical mediators (kairomones, pheromones), natural substances of animal, plant and mineral origin, and micro-organisms. The sale of micro-organisms is authorized on the biocontrol product market, such as bacteria (*Bacillus* sp., *Pseudomonas* sp., *Streptomyces* sp., etc.), oomycota (*Pythium* sp.), virus (*Cydia pomonella granulosis* virus, *Pepino mosaic virus*, etc.) and fungi (*Ampelomyces* sp., *Candida* sp., *Trichoderma* sp., etc.) [22]. AMF are not included among biocontrol products, although numerous studies have highlighted the ability of AMF to induce resistance in plants to biotic stresses, through the stimulation of plant defense mechanisms (Figure 2).

### 4.1. AMF-Induced Plant Protection against Pathogens

As Comby et al. [172] summarized in their review, mycorrhizal symbiosis can protect plants against a wide panel of plant pathogens, such as virus, bacteria, phytoplasma, fungi and pests by inducing several biochemical and molecular mechanisms. Firstly, improved photosynthesis and plant nutrition enhancement can help mycorrhized plants to overcome pathogen attacks [145,173]. Secondly, the extra-radical mycelium of AMF modulates the microbiota around the roots by competing spatially and nutritionally with belowground pathogenic microorganisms [174]. The mycorrhizal extra-radical mycelium also stimulates the activity of microorganisms, such as PGPR [175] (e.g., nitrogen-fixing bacteria) [176] and phosphate-solubilizing bacteria [177] that are beneficial to the plant in competition with belowground pathogenic microorganisms. Between plants of the same or different species, the AMF can form a mycelial network (common mycorrhizal network or CMN), which enables several sources of nutrients to be exchanged over long distances [178], and transfer of signal molecules between plants [179] or the induction of membrane depolarization [180]. For example, a mycorrhized plant attacked by a caterpillar or a necrotrophic fungus [181,182] warns its neighbors through the CMN, which can then activate their own defense mechanisms [179,183] in anticipation of a future attack.

### 4.2. Mycorrhiza-Induced Resistance

During the first interactions between a plant and an AMF, various MAMPs (microbe-associated molecular patterns) secreted by AMF are recognized by the plant, leading to a local plant immune response in the plant roots known as MAMP-triggered immunity (MTI) [184,185] (Figure 2). This immune response is associated with salicylic acid (SA) production, leading to the generation of long-distance signals moving along the vascular tissues and responsible for a transient systemic priming of SA-dependent defenses in other parts of the plant [186].

However, high levels of SA in root tissues are not favorable for the establishment of mycorrhizal symbiosis [187]. In response, AMF release effectors, which after recognition by the plant, induce ABA production in the roots [188]. ABA displays local immune suppressive effects, inhibiting SA-related defense mechanisms and resulting in root colonization. In addition, ABA could transit via the xylem from the roots to the aerial parts and activate cell-wall defense mechanisms, useful for protecting plants against aerial diseases [189].

Once the symbiosis is well established, a protection against pathogens can be observed in distal parts of the plants [190]. This protection is the result of the mycorrhiza-induced resistance (MIR) [20], due to the induction of a set of systemic defense mechanisms throughout the plant by AMF located in the roots [186] (Figure 2). The split-root system experiment by Cordier et al. [191] confirmed the systemic nature of MIR by inoculating half the roots of tomato plants with the AMF *F. mosseae,* and infecting the other half with the oomycete *Phytophthora nicotianae* var. *parasitica*. Mycorrhization induced resistance in the non-mycorrhized roots, which resulted in a reduction in root necrosis as well as a significant reduction in the development of the internal and external mycelium of the pathogenic fungus. This protection has been linked to the accumulation of non-esterified pectins and PR1a (pathogenesis-related protein 1) defense proteins in the cell walls, preventing penetration of the pathogen [192].

MIR relies on hormonal signaling. Depending on the tripartite system plant/pathogen/AMF, MIR could be associated with systemic-acquired resistance (SAR)-like priming of SA-dependent genes, but more often coincides with an induced resistance similar to the induced systemic resistance (ISR) priming of jasmonic acid (JA)-dependent genes [189]. Moreover, mycorrhizal symbiosis increases phosphate uptake and the transport of photosynthesis products from the leaves to roots [193], which modify the composition of root exudates. Enriched root exudates permit the recruitment of rhizobacteria and stimulate the development of the mycorrhizosphere [190]. Signaling compounds released by the selected bacteria (including *Pseudomonas* and *Burkholderia* strains) are recognized by the plant, which generates long-distance signals that prime JA- and ethylene-dependent plant defenses, characteristic of the ISR [194,195,196,197].

These plant hormones, whose production is modulated in mycorrhizal plants, induce a variety of defense mechanisms protecting against different crop pests and diseases. The JA- and ethylene-dependent defense appeared to protect mycorrhized plants against necrotrophic organisms [196,198], chewing insects [198,199] and to a lesser extent, against hemi-biotrophic pathogens [200]. The defense mechanisms are characterized by the upregulation of defense genes, such as lipoxygenase D gene (*LOXD*) and allene oxide cyclase gene (*AOC*) [201], the stimulation of polyphenol oxidase (PPO), phenylalanine ammonia lyase (PAL) or β-1,3 glucanase activities [192,201,202,203], the accumulation of phenolic compounds [203], PR1a protein, callose and pectin at the site of attempted penetration by the pathogen [192,201,202,203]. Against biotrophic pathogens, JA- and ethylene-dependent defense efficacy is more fluctuating. The SA-dependent defenses are known to be effective against hemi-biotrophic and biotrophic pathogens [186] and are characterized by the upregulation of defense genes enabling the production and accumulation of PR (pathogenesis-related) proteins [182], ROS [204], cell-wall reinforcing compounds (callose and phenolic compound deposition) [192,205], and the activation of the phenylpropanoid pathway in mycorrhized plants [206].

Thanks to the aforementioned abilities to strengthen the plant, leading to the set-up of plant protection against biotic stresses via MIR mechanisms, it can be argued that AMF inoculums can constitute an alternative to chemical phytosanitary products in controlling against a wide array of different pests and pathogens. However, numerous factors, specific to AMF or from the external environment, could temper these advantages, mainly observed in laboratory-controlled conditions.

### 4.3. Limits to the AMF-Inoculum Application as Biocontrol Agents

The efficacy of mycorrhizal protection depends on various factors. Firstly, mycorrhizal protection rate depends on the AMF species. Mustafa et al. [207] demonstrated that different AMF strains (*F. mosseae*, *R. irregularis* and Solrize^®^ (Burnley, UK) inoculum (mixtures of various *Glomus* sp.)) confer different rates of protection against powdery mildew in wheat. It has also been shown that the effectiveness of mycorrhizal inoculation depends on the plant species or varieties. Mora-Romero et al. [208] observed variable rates of protection through symbiosis with *R. irregularis* in tomato inoculated by *Xanthomonas campestris* and pea inoculated against *Sclerotinia sclerotiorum*. Similarly, Campo et al. [209] showed different rates of protection in twelve different rice cultivars against the rice blast fungus (*Magnaporthe oryzae*) in response to root inoculation with *F. mosseae* or *R. irregularis*. Then, few data are available on the effectiveness of AMF inoculums in inducing protection against aerial plant diseases [172]. Indeed, the majority of studies concern plant protection mediated by AMF against soilborne diseases [172,210]. There is a real need to explore more widely the relevance of AMF inoculum as a biocontrol tool against foliar diseases.

Additionally, plant breeding played a major role in modulating the symbiosis between AMF and the species and cultivars used in agriculture. Several studies have shown that old varieties and even ancestral wheat species roots are more colonized by AMF [211,212]. Hetrick et al. [212] explained that modern wheat varieties bred from the 1990s onwards were less sensitive to mycorrhizal symbiosis. Sawers et al. [213] argued that the plant breeding of modern wheat cultivars has improved their ability to extract more phosphate from the soil, thereby reducing their need to form a symbiosis with AMF. In addition, Parvin et al. [214] observed that modern rice cultivars formed a symbiosis with a lower diversity of AMF strains. Plant breeding, which has been practiced intensively for several decades, may therefore be a limiting factor for the effectiveness of inoculums as a biocontrol product.

Then, the choice of inoculum composition is important to obtain efficient protection against plant pests and diseases. The biocontrol effectiveness of commercial inoculums based on non-endemic AMF strains could be questionable. Commercial mycorrhizal inoculums on the market are composed of cosmopolitan AMF strains with low genetic diversity and are not necessarily adapted to the soil and climatic conditions of the regions in which they are used [215,216]. Elliott et al. [217] explained that, particularly in wheat, root colonization by commercial AMF propagules can be significant, but does not systematically increase nutrient uptake, thus not providing the biofertilizer effect for the crop targeted by the commercial mycorrhizal treatment. Consequently, several studies attested that the design and use of a consortia of AMF strains isolated from autochthonous soils would be more relevant and effective in protecting local crops. Indeed, by being better adapted to local conditions [218,219], endemic AMF strains can allocate more nutrients to mycorrhized plants, induce greater above-ground biomass [218,220], and increase the production of phenolic compounds useful for resistance to biotic stresses [221].

Furthermore, AMF from different clades can develop differently depending on favorable or unfavorable environmental conditions [222] by adopting completely divergent resource-use strategies (R or K strategies), also known as “Life History Strategies” (LHS) [223,224,225]. For example, AMF from the *Glomeraceae* family allocate resources to grow mainly inside roots, forming structures such as arbuscules, vesicles, hyphae [226], reproducing quickly [227] and sporulating abundantly [228]. De facto, *Glomeraceae* can develop even under an unstable environment (R strategies) [223]. Compared with *Glomeraceae*, *Gigasporaceae* grow slowly, produce more extra-radical structures [229,230], no vesicles and propagate mainly with spores [225,226]. *Gigasporaceae* prefer a stable environment (K strategists) [223]. Like the AMF *Gigasporaceae,* the *Acaulosporaceae* also grow slowly and have limited spore viability [231]. Consequently, it turns out that some AMF species used in commercial mycorrhizal inoculums could be invasive. Basiru and Hijri [232] reported that commercial inoculums are produced from strains of generic species (*F. mosseae*, *R. irregularis*, etc.), which can be considered invasive, as a result of their rapid development (R strategy) to the detriment of local AMF strains. As they have a faster reproductive capacity, they can also compete for nutritional resources with the native AMF populations [216]. The protective effect against pests and diseases induced by inoculated AMF could be considered relative if native soil biodiversity is compromised by the addition of invasive AMF strains. Conversely, if the inoculated strains are not capable of developing there because of their LHS, there is no need to select this inoculum, particularly when seeking protection against plant pests and diseases. In summary, the choice of local AMF strains could probably be the most relevant for environmentally-friendly usage and reliable plant protection [233]. However, Lutz et al. [234] have shown an interest in using invasive AMF strains in their analyses of the modulation of the soil microbiome in the situation of a significant supply of AMF inoculum to maize crops. The SAF22 strain, that they used to inoculate the maize plants, has largely competed with local AMF strains, but also with strains of edaphic fungal pathogens (*Olpidium* sp. *Cladosporium* sp., *Mycochaetophora* sp., etc.), providing maize plants indirectly with root protection.

Also, it should be noted that certain agronomic practices reduce the development and activity of AMF. For example, agricultural inputs such as the use of fungicides [235,236], and nitrogen and phosphate fertilization has often been observed to reduce the abundance and diversity of AMF in soils and the development of mycorrhization [237,238,239,240,241]. Soil ploughing disrupts soil structure [242,243], where *Glomeraceae* seem to be more resilient compared to *Gigasporaceae* [244,245]. In addition to AMF inoculation application, agronomic practices therefore need to be adapted in order to benefit from the positive effects of mycorrhization, and consequently take advantage of the protective potential of mycorrhizal inoculums.

## 5. AMF Inoculum Production

Jakobsen et al. [93] estimated that 80% of the conventional use of chemical phosphate fertilizers in the field could be offset by mycorrhizal inoculation. In this context, AMF have been used in agriculture, since the 1990s [246], as biostimulants [107,108]. Today, the production and sale of AMF inoculums represent a booming market all over the world (Table 2). The worldwide size of this market is projected to exceed USD 620 million by 2025 [247,248]. Producers are found mainly in Europe (United Kingdom, France, Germany, Italy, Czech Republic, Spain, Austria, Estonia, Switzerland and the Netherlands), North America (Canada and USA) and Asia (India and China, mainly) (Table 2). To a lesser degree, AMF-inoculum producers can be found in Central and South America (Mexico, Argentina, Brazil, Colombia) and Africa (e.g., Kenya) [12] (Table 2). According to Chen et al. [12], in 2018, AMF inoculums are mainly used in the sectors of horticulture, gardening and landscaping, agriculture and forestry. To a much lesser extent, AMF inoculums are found in soil remediation, golf course maintenance, renaturation of deteriorated soils, roof planting and research sectors [12]. According to Basiru et al. [248], commercial inoculums can contain one or more AMF species, including mostly *R. irregularis*, *F. mosseae*, and to a lesser extent *Claroideoglomus etunicatum*, *R. aggregatum*, *R. clarus*, *R. iranicus* and *Septoglomus deserticola*. They come in the form of fragments of mycorrhized roots, spores or mycorrhizal filaments, mixed with granules, powders, or in the form of a liquid solution, or in seed coatings [249], sometimes mixed with ectomycorrhizal fungi or PGPR. According to the same study [248], 60% of inoculums were formulated as powders, produced under *in vivo* conditions, and often composed of several species; 29% of inoculums were in the form of liquid inoculums, produced under *in vitro* conditions and preferably composed of a single AMF species. 

Estimating the economic impact of AMF inoculums as biostimulants is not widely discussed in the scientific literature. For example, Hijri [97] demonstrated that the use of AMF inoculums was affordable in field trials in potatoes, because the profit from harvesting was greater than the cost of using AMF inoculum (US$135 per hectare). Similarly, Tawaraya et al. [250] demonstrated that inoculation of Welsh onions (*Allium fistulosum* L.) with AMF was significantly more economically cost-effective (USD 2285 per ha) than conventional superphosphate fertilization (USD 5659 per ha). Furthermore, it has been observed that AMF can also help with phosphate fertilization reduction over several years, reducing the cost of using conventional phosphate fertilizers. In a rotation of maize and horse gram (year 1) and rice (year 2), Maiti et al. [251] demonstrated that the rice crop required 33% less phosphate input thanks to the AMF. Finally, the major economic advantage of using mycorrhizal inoculum lies in the fact that, unlike chemical inputs, it is not necessary to apply them every year in the field, provided that cultivation practices are compatible with the development of a mycelium network.

**Figure 3:**
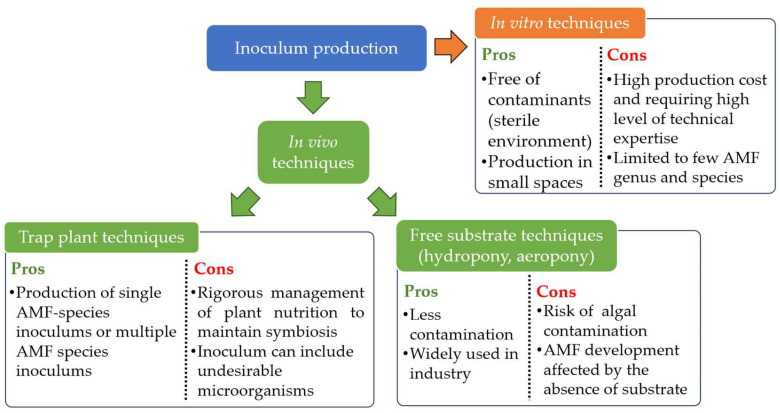
Pros and cons of the different techniques used to produce AMF inoculum.

As mentioned previously, AMF are therefore obligate biotrophs [27]. This characteristic prohibits their multiplication alone on the standard culture media used in microbiology, as Jones [252], Gallaud [253] and Peyronel, as cited in Harley [254], tried unsuccessfully. AMF inoculums can only be produced using whole plants or transformed live plant roots [255]. Numerous methods are regularly used for the mass production of AMF inoculums (Figure 3). The first is the “classic” method based on the production of a monospecific or multispecific inoculum from plants growing in bags, pots or beds filled with sterilized substrate and carried out mostly in greenhouse conditions [128]. AMF are multiplied in contact with the roots of trap plants that are easily mycorrhized, such as sorghum, maize, leek, clover, onion [256,257,258]. After production, the roots containing AMF propagules can be dried and reused, and the substrate sieved and/or decanted to recover the spores [259]. The produced substrate, enriched with AMF propagules, can also be used as AMF inoculum [260]. This method requires ongoing management of plant nutrition, and is not immune to any contamination already present in the substrate [259].

Since the end of the 1970s, other techniques have been developed to enable mycorrhizal inoculums to be produced in conditions of lower or non-contamination by other microorganisms. Some techniques, free of substrate, are using plants whose roots are bathing in a static nutrient solution (hydroponic cultivation) [261], located in a flowing nutrient solution (nutrient flow systems) [262,263] or sprayed with a nutrient solution (aeroponic cultivation) [264,265]. These methods are widely used nowadays at industrial scale [259]. However, contamination with algae and a lack of support for AMF can affect production [128]. Finally, techniques for growing AMF inoculum in *in vitro* conditions do exist. They use either Ri T-DNA root-transformed organs [266] placed into bioreactors comprising a solid support [267], airlift bioreactor [268] or small containers [269]. Whole plants can also be used with aerial organs outside a Petri dish while AMF and roots are in the Petri dish filled with a solid medium [270,271]. These *in vitro* techniques are performed in completely sterile conditions and should make it possible to produce large quantity, high quality and contaminant-free AMF inoculums [259,272]. Also, these techniques enable production to be carried out in much more confined spaces [272]. However, the high level of technical expertise required to operate and maintain these techniques on an industrial scale means that production costs remain relatively high [259,272]. Finally, these *in vitro* techniques are limited to a few AMF species [272], from the genus *Rhizophagus* sp. and few *Gigasporaceae* species [272].

The production of AMF inoculums therefore concerns an increasingly developed market, with a whole range of production techniques used by a multitude of companies around the world. These economic players could be involved in AMF inoculum production for the biocontrol market.

## 6. Conclusions and Future Perspectives

AMF are symbiotic living micro-organisms, members of a lineage of fungi whose great diversity has yet to be explored. Overall, AMF provide nutritional benefits to the host plants which can improve plant growth and yields in unfavorable conditions. Nowadays, AMF are then used as biostimulant products in the form of inoculums of different compositions and natures, whose diversity is often limited to the same cosmopolitan mycorrhizal families (mainly from *R. irregularis*. and *F. mosseae*). These inoculums are produced using efficient but costly techniques, and are used by dozens of companies and research centers around the world, mainly located in Europe, Northern America, India and China. Furthermore, AMF are able to induce a wide range of systemic defense mechanisms, which are effective in protecting plants against a variety of pathogens and pests with different lifestyles (biotrophic, hemi-biotrophic, necrotrophic), responsible for root and foliar diseases. The induction of defense mechanisms is dependent on plant hormones such as SA, JA and ethylene, whose production is modulated by mycorrhization. Thus, the use of AMF represents an interesting alternative solution for plant protection thanks to the induced plant resistance via MIR. The use of mycorrhizal inoculums could then constitute a new additional agronomic technique to reduce the use of phytosanitary products [172,273]. AMF can be considered as an ecosystemic service [189] and could complement innovative protective strategies in the development of more sustainable agriculture [108,274,275]. However, a number of factors, both specific to AMF and external to it (plants or environment), can modulate the protective efficacy mediated by AMF. Therefore, more research is required on plant diseases, to determine the protective effectiveness against biotic stress. Furthermore, the impact of AMF inoculum on the immediate environment, in particular on the soil microbiota must be investigated. In addition, as Lekberg and Helgason [276] pointed out, many biotic and abiotic factors in the field modulate the effects observed with mycorrhizal inoculums obtained under controlled conditions. This means that this research will have to be carried out under uncontrolled conditions, i.e., through the implementation of many more experimental field trials.

## Figures and Tables

**Table 1:** Current and evolving taxonomic classification of arbuscular mycorrhizal fungi.

Class	Order	Family	Genera	Reference
Archaeosporomycetes	Archaeosporales	*Ambisporaceae* *Archaeosporaceae* *Geosiphonaceae* *Polonosporaceae*	*Ambispora* *Archaeospora* *Geosiphon* *Polonospora*	Confirmed taxa (2013) [39] Formerly in Intraspora (2013) [39] Confirmed taxa (2013) [39] Recent taxa (2021) [41]
Glomeromycetes	Diversisporales	*Acaulosporaceae*	*Acaulospora*	Merged with *Kuklospora* (2013) [39]
		*Diversisporaceae*	*Corymbiglomus* *Desertispora* *Diversispora* *Otospora* *Redeckera* *Sieverdingia* *Tricispora*	Reclassified taxa (2012) [42] Recent taxa (2018) [43] Confirmed taxa (2013) [39] Uncertain taxa (2013) [39] Confirmed taxa (2013) [39] Reclassified taxa (2019) [44] Uncertain taxa (2013) [39]
		*Gigasporaceae*	*Bulbospora* *Cetraspora* *Dentiscutata* *Fuscutata* *Gigaspora* *Intraornatospora* *Paradentiscutata* *Racocetra* *Scutellospora*	Recent taxa (2014) [45] Uncertain taxonomy (2013) [39], formerly in *Racocetraceae* (2011) [40] Formerly in *Quatunica* (2013) [39] Formerly in *Dentiscutata* (2013) [39] Confirmed taxa (2013) [39] Uncertain taxonomy (2013) [39], formerly in *Intraometosporaceae* (2011) [40] Uncertain taxonomy (2013) [39], formerly in *Intraometosporaceae* (2011) [40] Confirmed taxa (2013) [39] Formerly in *Orbispora* (2013) [39]
		*Pacisporaceae*	*Pacispora*	Confirmed taxa (2013) [39]
		*Sacculosporaceae*	*Sacculospora*	Uncertain taxonomy (2013) [39]
	Entrophosporales ^1^	*Entrophosporaceae* ^2^	*Entrophospora* ^3^	^1^ Recent taxa [38], ^2^ uncertain taxonomy (2013) [39], ^3^ formerly in *Claroideoglomus*
	Glomerales	*Glomeraceae*	*Complexispora* *Dominikia* *Epigeocarpum* *Funneliformis* *Funneliglomus* *Glomus* *Halonatospora* *Kamienskia* *Microdominikia* *Microkamienskia* *Nanoglomus* *Oehlia* *Orientoglomus* *Rhizoglomus* *Rhizophagus* *Sclerocarpum* *Sclerocystis* *Septoglomus* *Silvaspora*	Recent taxa (2023) [46] Recent taxa (2015) [47] Recent taxa (2021) [41] Confirmed taxa in 2013 [39] Recent taxa (2019) [48] Formerly in Simiglomus (2013) [39] Recent taxa (2018) [49] Recent taxa (2015) [47] Recent taxa (2018) [50] Recent taxa (2019) [51] Recent taxa (2019) [52] Recent taxa (2019) [51] Recent taxa (2019) [52] Recent taxa (2014) [53] Reclassified taxa (2012) [42] Recent taxa (2019) [54] Reclassified taxa (2012) [42] Formerly in Viscospora (2013) [39] Recent taxa (2021) [41]
Paraglomeromycetes	Paraglomerales	*Paraglomerales*	*Innospora*	Recent taxa (2017) [55]
			*Paraglomus*	Confirmed taxa (2013) [39]
		*Pervetustaceae*	*Pervetustus*	Recent taxa (2017) [55]

**Table 2:** Examples of AMF-inoculum producers and companies grouped by country (2024).

Countries	Examples of AMF-Inoculum Producers
Austria	Biofa (Graz); GEFA Produkte^®^ Fabritz GmbH (mother company in Krefeld, Germany)
Belgium	Glomeromycota IN vitro COllection (GINCO) (Louvain-la-Neuve); Plantura (Bocholt)
Canada	Premier Tech Ltd. (Rivière-du-Loup, QC); Canadian Collection of Arbuscular Mycorrhizal Fungi (CCAMF) (Ottawa, ON); Glomeromycota IN vitro COllection (GINCO) (Ottawa, ON); Lallemand Inc. (Montreal, QC); Mikro-Tek Inc. (Timmins, ON)
Chile	Biosim (Chiu Chiu); Idemitsu Kosan Co. (mother company in Tokyo, Japan); Ecological Resources; Inc./Oikos (mother company in Elizabeth, PA, United States of America)
China	Guangdong Microbial Culture Collection Center (GDMCC) (Guangzhou); Weifang Yuedong International Trade Co., Ltd. (Weifang); Weifang Yuexiang Chemical Co., Ltd. (Weifang); Zhejiang Shijia Technology Co., Ltd. (Zhejiang Sheng)
Czech Republic	Symbiom SRO (Lanškroun)
Estonia	Mikskaar (Tallinn)
France	Agronutrition (Carbonne); IF tech (Les Ponts-de-Cé); InoculumPlus (Bretenière); International Bank for the Glomeromycota (IBG) (Dijon); MycAgro (Bretenière); Terra fertilis (Caen); Mycoterroir (Montpellier); Semences de France (La Chapelle-d’Armentières)
Germany	Agromyc-Merck GmbH (Hamburg); Biofa GmbH (Münsingen); BioMyc™ (Brandenburg an der Havel); Inoq GmbH (Schnega); Mykolife (Gersthofen); Symplanta GmbH & Co. KG (Darmstadt)
India	AgriLife (Hyderabad); Ambika Biotech (Mandsaur); Anand Agro Care (Maharashtra); Biotrack Technology Pvt. Ltd. (Chennai); Centre for Mycorrhizal Culture Collection (CMCC) (New Delhi); Cosme Biotech (Panaji); Dr. Rajan Laboratories (Tambaram); GreenMax AgroTech (Adikaratti); Kiran Chemicals (Lucknow); Katyayani (Katyayani); Majestic Agronomics Pvt. Ltd. (Janakpuri); Neesa Agritech Private Limited (Changodar); Neologie Bio Innovations|Private Limited (Rampur); PHMS Technicare Private Limited (Gujarat); Privi Life Sciences (Mumbai); Sikko Industries (Vejalpur); Sundaram Overseas Operation (Mumbai); T. Stanes & Company Limited (Coimbatore); TARI Biotech (Thanjavur); TERI (New Delhi); ManiDharma Biotech Pvt. Ltd. (Chennai)
Israel	Groundwork AG (Mazor)
Italy	Agritech Store sas (Mori); Agribios (Villafranca Padovana); Hello nature (Biandrate); Sacom (Turin); CCS Aosta S.r.l (Villair-Amérique)
Japan	Central Glass Co., Chemicals Section (Tokyo); Idemitsu Kosan Co. Ltd. (Tokyo); Kyowa Hakko Bio Co. Ltd. (Nakano-ku Tokyo)
Kenya	Dudutech (Naivasha)
Malaysia	Agri Hi-Tech Sdn (Nilai); N-Viron Sdn Bhd (Klang)
Mexico	Instituto Nacional de Investigación Forestales Agrícolas y Pecuario (INIFAP) (Ciudad de Mexico); Biofabrica Siglo XXI (Mor); Biokrone (Celaya); Biomic (Ciudad de Mexico); OBA (Autlán de Navarro); Agrovergel (El vergel)
Netherlands	BioTabs Organic Fertilizers (Den Haag); Global Horticare (Nijmegen); Koppert (Berkel en Rodenrijs)
Spain	Agrotechnologias Naturales (Atens) (Tarragona); Biohorti SLU (Teia); Mycosym Trition S (Riogordo); Mycovitro (Granada); Odd Distributions (Pedreguer)
Poland	Mykoflor Wáodzimierz SzaáaĔski (Końskowola)
Portugal	Asfertglobal (Várzea)
United Kingdom	Biological Crop Protection Ltd. (Chichester); Crop Intellect Ltd. (Lincoln); PlantWorks Ltd. (Sittingbourne); Zander Corporation (London)
United States of America	AgBio, Inc. (Del Rio, CA); AgroScience Solutions LLC (Bakersfield, CA); Albright Seed Co./S & S Seeds (Carpinteria, CA); Becker Underwood (BASF) (Ames, IA); Bio-Organics (New Hope, PA); BioScientific, Inc. (Avondale, AZ); EcoLife Corporation (Everett, WA); First Fruits, LLC (Prescott, WA); Fungi Perfecti (Olympia, WA); Gro-Power (Chino, CA); Helana Agri-Entreprises LLC (Collierville, TN); Woodridge International (New Haven, CT); Horticultural Alliance, Inc (Sarasota, FL); International Collection of Vesicular Arbuscular Mycorrhizal Fungi (INVAM) (Lawrence, KS); JH Biotech (Ventura, CA); MYCSA Ag, Inc. EUA (Brownsville, TX); Mycorrhizal Applications (Grants Pass, OR); Pathway BioLogic LLC (Plant City, FL); Poulenger USA, Inc. (Lakeland, FL); Purely Organic Products LLC (Portsmouth, NH); Reforestation Technologies International (Gilroy, CA); ROOTS, Inc. (Vancouver, WA); Shemin Garden LLC (Clovis, CA); Sustane Natural Fertilizer (Cannon Falls, MN); Tainio Biologicals Inc. (Spokane, WA); The Tree Doctor (San Diego, CA); Tree Pro (Phoenix, AZ); Valent BioSciences (Libertyville, IL); Ecological Resources, Inc./Oikos (Elizabeth, PA)
Switzerland	Swiss culture collection of Arbuscular Mycorrhizal Fungi (Bern); Vegalab S.A. (Zug)

## Data Availability

The original contributions presented in the study are included in the article, further inquiries can be directed to the corresponding author.

## References

[1] De Jaeger C., Cherin P., Fraoucene N., Voronska E. (2012). Place, Intérêt et Danger Des Produits Phytosanitaires. Med. Longevite.

[2] United Nations Population Division World Population Prospects. https://population.un.org/wpp/.

[3] Boedeker W., Watts M., Clausing P., Marquez E. (2020). The Global Distribution of Acute Unintentional Pesticide Poisoning: Estimations Based on a Systematic Review. BMC Public Health.

[4] Potts S.G., Imperatriz-Fonseca V., Ngo H.T., Biesmeijer J.C., Breeze T.D., Dicks L.V., Garibaldi L.A., Hill R., Settele J., Vanbergen A.J. (2016). Résumé à l’Intention des Décideurs du Rapport d’Evaluation de la Plateforme Intergouvernementale Scientifique et Politique sur la Biodiversité et les Services Écosystémiques Concernant les Pollinisateurs, la Pollinisation et la Production Alimentaire.

[5] Karadimitriou N., Cheru F., Wondimu A., Yacobi H., Eyob A., Belay F., Temesgen T., Eyana S., Yoseph S. (2017). Global Assessment of the Impact of Plant Protection Products on Soil Functions and Soil Ecosystems.

[6] Kaur H., Garg H. (2014). Pesticides: Environmental Impacts and Management Strategies. Pestic. Toxic Asp..

[7] Assouline G. (1989). L’évolution Technologique de l’industrie Des Phytosanitaires: Quelles Interactions Avec l’agriculture?. Économie Rural..

[8] European Parliament (2022). Proposal for a Regulation of the European Parliament and of the Council on the Sustainable Use of Plant Protection Products and Amending Regulation (EU) 2021/2115.

[9] Food and Agriculture Organization of the United Nations Integrated Pest Management (IPM)|Pest and Pesticide Management|IPM and Pesticide Risk Reduction. https://www.fao.org/pest-and-pesticide-management/ipm/integrated-pest-management/en/.

[10] European Parliament (2018). Regulation (EU) 2018/848 of the European Parliament and of the Council of 30 May 2018 on Organic Production and Labelling of Organic Products and Repealing Council Regulation (EC) No 834/2007.

[11] du Jardin P. (2015). Plant Biostimulants: Definition, Concept, Main Categories and Regulation. Sci. Hortic..

[12] Chen M., Arato M., Borghi L., Nouri E., Reinhardt D. (2018). Beneficial Services of Arbuscular Mycorrhizal Fungi—From Ecology to Application. Front. Plant Sci..

[13] McNear D.H. (2013). The Rhizosphere—Roots, Soil and Everything In Between. Nat. Sci. Educ..

[14] Ryder L.S., Harris B.D., Soanes D.M., Kershaw M.J., Talbot N.J., Thornton C.R. (2012). Saprotrophic Competitiveness and Biocontrol Fitness of a Genetically Modified Strain of the Plant-Growth-Promoting Fungus *Trichoderma hamatum* GD12. Microbiology.

[15] Harman G.E., Howell C.R., Viterbo A., Chet I., Lorito M. (2004). Trichoderma Species—Opportunistic, Avirulent Plant Symbionts. Nat. Rev. Microbiol..

[16] Attia M.S., Abdelaziz A.M., Al-Askar A.A., Arishi A.A., Abdelhakim A.M., Hashem A.H. (2022). Plant Growth-Promoting Fungi as Biocontrol Tool against Fusarium Wilt Disease of Tomato Plant. J. Fungi.

[17] Abbott L.K., Robson A.D. (1977). Growth Stimulation of Subterranean Clover with Vesicular Arbuscular Mycorrhizas. Aust. J. Agric. Res..

[18] Union Européenne Regulation (EU) 2019/1009 of the European Parliament and of the Council of 5 June 2019 Laying down Rules on the Making Available on the Market of EU Fertilising Products and Amending Regulations (EC) No 1069/2009 and (EC) No 1107/2009 and Repealing Regulation (EC) No 2003/2003. https://eur-lex.europa.eu/legal-content/EN/TXT/?uri=OJ:L:2019:170:TOC.

[19] Israel A., Langrand J., Fontaine J., Lounès-Hadj Sahraoui A. (2022). Significance of Arbuscular Mycorrhizal Fungi in Mitigating Abiotic Environmental Stress in Medicinal and Aromatic Plants: A Review. Foods.

[20] Pozo M.J., Verhage A., García-Andrade J., García J.M., Azcón-Aguilar C., Azcón-Aguilar C., Barea J., Gianinazzi S., Gianinazzi-Pearson V. (2009). Priming Plant Defence Against Pathogens by Arbuscular Mycorrhizal Fungi. Mycorrhizas—Functional Processes and Ecological Impact.

[21] IBMA Home Page IBMA-GLOBAL International Biocontrol Manufacturers. https://ibma-global.org/.

[22] Ministère de l’Agriculture et de la Souveraineté Alimentaire (2024). Liste des Produits Phytopharmaceutiques de Biocontrôle, au Titre des Articles L.253-5 et L.253-7 du Code Rural et de la Pêche Maritime.

[23] Frank A.B. (1885). Ueber Die Auf Wurzelsymbiose Beruhende Ernährung Gewisser Baüme Durch Unterirdische Pilze. Ber. Dtsch. Bot. Ges..

[24] von Nägeli C.W. (1842). Pilze Im Innern von Zellen. Linnaea.

[25] Tisserant E., Malbreil M., Kuo A., Kohler A., Symeonidi A., Balestrini R., Charron P., Duensing N., Frei Dit Frey N., Gianinazzi-Pearson V. (2013). Genome of an Arbuscular Mycorrhizal Fungus Provides Insight into the Oldest Plant Symbiosis. Proc. Natl. Acad. Sci. USA.

[26] Garcia-Garrido J.M., Antonio Ocampo J., Garcia-Romera I. (2002). Enzymes in the Arbuscular Mycorrhizal Symbiosis.

[27] Bonfante P., Perotto S. (1995). Tansley Review No. 82. Strategies of Arbuscular Mycorrhizal Fungi When Infecting Host Plants. New Phytol..

[28] Smith S.E., Read D. (2008). The Symbionts Forming Arbuscular Mycorrhizas. Mycorrhizal Symbiosis.

[29] Wang Y., He X., Yu F. (2022). Non-Host Plants: Are They Mycorrhizal Networks Players?. Plant Divers..

[30] Redecker D., Kodner R., Graham L.E. (2000). Glomalean Fungi from the Ordovician. Science.

[31] Krüger M., Krüger C., Walker C., Stockinger H., Schüßler A. (2012). Phylogenetic Reference Data for Systematics and Phylotaxonomy of Arbuscular Mycorrhizal Fungi from Phylum to Species Level. New Phytol..

[32] Corradi N., Kuhn G., Sanders I.R. (2004). Monophyly of β-Tubulin and H+-ATPase Gene Variants in *Glomus Intraradices*: Consequences for Molecular Evolutionary Studies of AM Fungal Genes. Fungal Genet. Biol..

[33] Gollotte A., Van Tuinen D., Atkinson D. (2004). Diversity of Arbuscular Mycorrhizal Fungi Colonising Roots of the Grass Species *Agrostis Capillaris* and *Lolium Perenne* in a Field Experiment. Mycorrhiza.

[34] Helgason T., Fitter A.H., Young J.P.W. (1999). Molecular Diversity of Arbuscular Mycorrhizal Fungi Colonising *Hyacinthoides Non-Scripta* (Bluebell) in a Seminatural Woodland. Mol. Ecol..

[35] Wubet T., Weiß M., Kottke I., Teketay D., Oberwinkler F. (2004). Molecular Diversity of Arbuscular Mycorrhizal Fungi in *Prunus Africana*, an Endangered Medicinal Tree Species in Dry Afromontane Forests of Ethiopia. New Phytol..

[36] Schüßler A., Schwarzott D., Walker C. (2001). A New Fungal Phylum, the *Glomeromycota*: Phylogeny and Evolution. Mycol. Res..

[37] Wijayawardene N., Hyde K., Dai D., Sánchez-García M., Goto B., Saxena R., Erdoğdu M., Selçuk F., Rajeshkumar K.C., Aptroot A. (2022). Outline of Fungi and Fungus-like Taxa—2021. Mycosphere.

[38] Błaszkowski J., Sánchez-García M., Niezgoda P., Zubek S., Fernández F., Vila A., Al-Yahya’ei M.N., Symanczik S., Milczarski P., Malinowski R. (2022). A New Order, Entrophosporales, and Three New *Entrophospora* Species in Glomeromycota. Front. Microbiol..

[39] Redecker D., Schüßler A., Stockinger H., Stürmer S.L., Morton J.B., Walker C. (2013). An Evidence-Based Consensus for the Classification of Arbuscular Mycorrhizal Fungi (*Glomeromycota*). Mycorrhiza.

[40] Oehl F., Sieverding E., Palenzuela J., Ineichen K., da Silva G.A. (2011). Advances in *Glomeromycota* Taxonomy and Classification. IMA Fungus.

[41] Błaszkowski J., Niezgoda P., Meller E., Milczarski P., Zubek S., Malicka M., Uszok S., Casieri L., Goto B.T., Magurno F. (2021). New Taxa in *Glomeromycota*: *Polonosporaceae* Fam. Nov., *Polonospora* Gen. Nov., and *P. Polonica* Comb. Nov. Mycol. Prog..

[42] Goto B.T., Silva G.A., De Assis D.M.A., Silva D.K.A., Souza R.G., Ferreira A.C.A., Jobim K., Mello C.M.A., Vieira H.E.E., Maia L.C. (2012). *Intraornatosporaceae* (*Gigasporales*), a New Family with Two New Genera and Two New Species. Mycotaxon.

[43] Symanczik S., Al-Yahya’ei M.N., Kozłowska A., Ryszka P., Błaszkowski J. (2018). A New Genus, *Desertispora*, and a New Species, *Diversispora Sabulosa*, in the Family Diversisporaceae (Order Diversisporales, Subphylum Glomeromycotina). Mycol. Prog..

[44] Błaszkowski J., Niezgoda P., de Paiva J.N., da Silva K.J.G., Theodoro R.C., Jobim K., Orfanoudakis M., Goto B.T. (2019). *Sieverdingia* Gen. Nov., *S. Tortuosa* Comb. Nov., and *Diversispora Peloponnesiaca* Sp. Nov. in the Diversisporaceae (Glomeromycota). Mycol. Prog..

[45] Marinho F., Da Silva G.A., Ferreira A.C.A., Da Nóbrega Veras J.S., Da Sousa N.M.F., Goto B.T., Maia L.C., Oehl F. (2014). *Bulbospora Minima*, a New Genus and a New Species in the Glomeromycetes from Semi-Arid Northeast Brazil. Sydowia.

[46] Błaszkowski J., Yamato M., Niezgoda P., Zubek S., Milczarski P., Malinowski R., Meller E., Malicka M., Goto B.T., Uszok S. (2023). A New Genus, *Complexispora*, with Two New Species, *C. multistratosa* and *C. mediterranea*, and *Epigeocarpum japonicum* Sp. Nov. Mycol. Prog..

[47] Błaszkowski J., Chwat G., Góralska A., Ryszka P., Kovács G.M. (2015). Two New Genera, *Dominikia* and *Kamienskia*, and *D. disticha* Sp. Nov. in Glomeromycota. Nova Hedwig..

[48] Corazon-Guivin M.A., Mendoza A.C., Guerrero-Abad J.C., Vallejos-Tapullima A., Carballar-Hernández S., Da Silva G.A., Oehl F. (2019). *Funneliglomus*, Gen. Nov., and *Funneliglomus sanmartinensis*, a New Arbuscular Mycorrhizal Fungus from the Amazonia Region in Peru. Sydowia.

[49] Błaszkowski J., Niezgoda P., Goto B.T., Kozłowska A. (2018). *Halonatospora* Gen. Nov. with *H. Pansihalos* Comb. Nov. and *Glomus Bareae* Sp. Nov. (Glomeromycota; Glomeraceae). Botany.

[50] Błaszkowski J., Ryszka P., Kozłowska A. (2018). *Dominikia Litorea*, a New Species in the Glomeromycotina, and Biogeographic Distribution of *Dominikia*. Phytotaxa.

[51] Corazon-Guivin M.A., Cerna-Mendoza A., Guerrero-Abad J.C., Vallejos-Tapullima A., Carballar-Hernández S., da Silva G.A., Oehl F. (2019). *Microkamienskia* Gen. Nov. and *Microkamienskia Peruviana*, a New Arbuscular Mycorrhizal Fungus from Western Amazonia. Nova Hedwig..

[52] Corazon-Guivin M.A., Cerna-Mendoza A., Guerrero-Abad J.C., Vallejos-Tapullima A., Carballar-Hernández S., da Silva G.A., Oehl F. (2019). *Nanoglomus Plukenetiae*, a New Fungus from Peru, and a Key to Small-Spored Glomeraceae Species, Including Three New Genera in the “*Dominikia* Complex/Clades”. Mycol. Prog..

[53] Sieverding E., Da Silva G.A., Berndt R., Oehl F. (2014). *Rhizoglomus*, a New Genus of the *Glomeraceae*. Mycotaxon.

[54] Jobim K., Błaszkowski J., Niezgoda P., Kozłowska A., Zubek S., Mleczko P., Chachuła P., Kazue Ishikawa N., Goto B.T., Thines M. (2019). New Sporocarpic Taxa in the Phylum Glomeromycota: *Sclerocarpum Amazonicum* Gen. et Sp. Nov. in the Family *Glomeraceae* (Glomerales) and *Diversispora Sporocarpia* Sp. Nov. in the *Diversisporaceae* (Diversisporales). Mycol. Prog..

[55] Błaszkowski J., Kozłowska A., Crossay T., Symanczik S., Al-Yahya’ei M.N. (2017). A New Family, Pervetustaceae with a New Genus, *Pervetustus*, and *P. Simplex* Sp. Nov. (Paraglomerales), and a New Genus, *Innospora* with *I. Majewskii* Comb. Nov. (Paraglomeraceae) in the Glomeromycotina. Nova Hedwig..

[56] Steinkellner S., Lendzemo V., Langer I., Schweiger P., Khaosaad T., Toussaint J.P., Vierheilig H. (2007). Flavonoids and Strigolactones in Root Exudates as Signals in Symbiotic and Pathogenic Plant-Fungus Interactions. Molecules.

[57] Mayzlish-Gati E., De-Cuyper C., Goormachtig S., Beeckman T., Vuylsteke M., Brewer P.B., Beveridge C.A., Yermiyahu U., Kaplan Y., Enzer Y. (2012). Strigolactones Are Involved in Root Response to Low Phosphate Conditions in Arabidopsis. Plant Physiol..

[58] Akiyama K., Matsuzaki K.I., Hayashi H. (2005). Plant Sesquiterpenes Induce Hyphal Branching in Arbuscular Mycorrhizal Fungi. Nature.

[59] Besserer A., Puech-Pagès V., Kiefer P., Gomez-Roldan V., Jauneau A., Roy S., Portais J.C., Roux C., Bécard G., Séjalon-Delmas N. (2006). Strigolactones Stimulate Arbuscular Mycorrhizal Fungi by Activating Mitochondria. PLoS Biol..

[60] Tamasloukht B., Séjalon-Delmas N., Kluever A., Jauneau A., Roux C., Bécard G., Franken P. (2003). Root Factors Induce Mitochondrial-Related Gene Expression and Fungal Respiration during the Developmental Switch from Asymbiosis to Presymbiosis in the Arbuscular Mycorrhizal Fungus *Gigaspora Rosea*. Plant Physiol..

[61] Vierheilig H., Bago B., Albrecht C., Poulin M.J., Piché Y. (1998). Flavonoids and Arbuscular-Mycorrhizal Fungi. Adv. Exp. Med. Biol..

[62] Koske R.E. (1981). Multiple Germination by Spores of *Gigaspora Gigantea*. TBMS.

[63] Mosse B. (1959). The Regular Germination of Resting Spores and Some Observations on the Growth Requirements of an *Endogone* Sp. Causing Vesicular-Arbuscular Mycorrhiza. TBMS.

[64] Paszkowski U. (2006). A Journey through Signaling in Arbuscular Mycorrhizal Symbioses 2006. New Phytol..

[65] Maillet F., Poinsot V., André O., Puech-Pagés V., Haouy A., Gueunier M., Cromer L., Giraudet D., Formey D., Niebel A. (2011). Fungal Lipochitooligosaccharide Symbiotic Signals in Arbuscular Mycorrhiza. Nature.

[66] Genre A., Chabaud M., Timmers T., Bonfante P., Barker D.G. (2005). Arbuscular Mycorrhizal Fungi Elicit a Novel Intracellular Apparatus in *Medicago Truncatula* Root Epidermal Cells before Infection. Plant Cell.

[67] Genre A., Chabaud M., Faccio A., Barker D.G., Bonfante P. (2008). Prepenetration Apparatus Assembly Precedes and Predicts the Colonization Patterns of Arbuscular Mycorrhizal Fungi within the Root Cortex of Both *Medicago Truncatula* and *Daucus Carota*. Plant Cell.

[68] Brundrett M.C. (2002). Coevolution of Roots and Mycorrhizas of Land Plants. New Phytol..

[69] Jakobsen I., Rosendahl L. (1990). Carbon Flow into Soil and External Hyphae from Roots of Mycorrhizal Cucumber Plants. New Phytol..

[70] Parniske M. (2008). Arbuscular Mycorrhiza: The Mother of Plant Root Endosymbioses. Nat. Rev. Microbiol..

[71] Bago B., Pfeffer P.E., Shachar-Hill Y. (2000). Carbon Metabolism and Transport in Arbuscular Mycorrhizas. Plant Physiol..

[72] Bravo A., Brands M., Wewer V., Dörmann P., Harrison M.J. (2017). Arbuscular Mycorrhiza-Specific Enzymes FatM and RAM2 Fine-Tune Lipid Biosynthesis to Promote Development of Arbuscular Mycorrhiza. New Phytol..

[73] Lehmann A., Rillig M.C. (2015). Arbuscular Mycorrhizal Contribution to Copper, Manganese and Iron Nutrient Concentrations in Crops—A Meta-Analysis. Soil Biol. Biochem..

[74] Garcia K., Doidy J., Zimmermann S.D., Wipf D., Courty P.E. (2016). Take a Trip Through the Plant and Fungal Transportome of Mycorrhiza. Trends Plant Sci..

[75] Wipf D., Krajinski F., van Tuinen D., Recorbet G., Courty P.E. (2019). Trading on the Arbuscular Mycorrhiza Market: From Arbuscules to Common Mycorrhizal Networks. New Phytol..

[76] Govindarajulu M., Pfeffer P.E., Jin H., Abubaker J., Douds D.D., Allen J.W., Bücking H., Lammers P.J., Shachar-Hill Y. (2005). Nitrogen Transfer in the Arbuscular Mycorrhizal Symbiosis. Nature.

[77] Garcia K., Zimmermann S.D. (2014). The Role of Mycorrhizal Associations in Plant Potassium Nutrition. Front. Plant Sci..

[78] Watts-Williams S.J., Gill A.R., Jewell N., Brien C.J., Berger B., Tran B.T.T., Mace E., Cruickshank A.W., Jordan D.R., Garnett T. (2022). Enhancement of Sorghum Grain Yield and Nutrition: A Role for Arbuscular Mycorrhizal Fungi Regardless of Soil Phosphorus Availability. Plants People Planet.

[79] Chen S., Zhao H., Zou C., Li Y., Chen Y., Wang Z., Jiang Y., Liu A., Zhao P., Wang M. (2017). Combined Inoculation with Multiple Arbuscular Mycorrhizal Fungi Improves Growth, Nutrient Uptake and Photosynthesis in Cucumber Seedlings. Front. Microbiol..

[80] Aroca R., Porcel R., Ruiz-Lozano J.M. (2007). How Does Arbuscular Mycorrhizal Symbiosis Regulate Root Hydraulic Properties and Plasma Membrane Aquaporins in Phaseolus Vulgaris under Drought, Cold or Salinity Stresses?. New Phytol..

[81] Kakouridis A., Hagen J.A., Kan M.P., Mambelli S., Feldman L.J., Herman D.J., Weber P.K., Pett-Ridge J., Firestone M.K. (2022). Routes to Roots: Direct Evidence of Water Transport by Arbuscular Mycorrhizal Fungi to Host Plants. New Phytol..

[82] Kobae Y., Hata S. (2010). Dynamics of Periarbuscular Membranes Visualized with a Fluorescent Phosphate Transporter in Arbuscular Mycorrhizal Roots of Rice. Plant Cell Physiol..

[83] Javot H., Penmetsa R.V., Terzaghi N., Cook D.R., Harrison M.J. (2007). A *Medicago Truncatula* Phosphate Transporter Indispensable for the Arbuscular Mycorrhizal Symbiosis. Proc. Natl. Acad. Sci. USA.

[84] Bach E.M., Narvaez-Rivera G., Murray K., Bauer J.T., Hofmockel K.S. (2018). The Dynamic Life of Arbuscular Mycorrhizal Fungal Symbionts. Ecology.

[85] Bucher M., Wegmüller S., Drissner D. (2009). Chasing the Structures of Small Molecules in Arbuscular Mycorrhizal Signaling. Curr. Opin. Plant Biol..

[86] Wu Q.S., Xia R.X., Zou Y.N. (2008). Improved Soil Structure and Citrus Growth after Inoculation with Three Arbuscular Mycorrhizal Fungi under Drought Stress. Eur. J. Soil Biol..

[87] Rillig M.C. (2004). Arbuscular Mycorrhizae, Glomalin, and Soil Aggregation. Can. J. Soil Sci..

[88] Syamsiyah J., Herawati A., Mujiyo (2018). The Potential of Arbuscular Mycorrhizal Fungi Application on Aggregrate Stability in Alfisol Soil. Proceedings of the 4th International Conference on Sustainable Agriculture and Environment (4th ICSAE).

[89] Bowles T.M., Barrios-Masias F.H., Carlisle E.A., Cavagnaro T.R., Jackson L.E. (2016). Effects of Arbuscular Mycorrhizae on Tomato Yield, Nutrient Uptake, Water Relations, and Soil Carbon Dynamics under Deficit Irrigation in Field Conditions. Sci. Total Environ..

[90] Bender S.F., Conen F., Van der Heijden M.G.A. (2015). Mycorrhizal Effects on Nutrient Cycling, Nutrient Leaching and N2O Production in Experimental Grassland. Soil Biol. Biochem..

[91] Wu Q.-S., Huang Y.-M., Li Y., He X.-H. (2014). Contribution of Arbuscular Mycorrhizas to Glomalin-Related Soil Protein, Soil Organic Carbon and Aggregate Stability in Citrus Rhizosphere. Int. J. Agric. Biol..

[92] Agnihotri R., Sharma M.P., Prakash A., Ramesh A., Bhattacharjya S., Patra A.K., Manna M.C., Kurganova I., Kuzyakov Y. (2022). Glycoproteins of Arbuscular Mycorrhiza for Soil Carbon Sequestration: Review of Mechanisms and Controls. Sci. Total Environ..

[93] Jakobsen I. (1995). Transport of Phosphorus and Carbon in VA Mycorrhizas. Mycorrhiza: Structure, Function, Molecular Biology and Biotechnology.

[94] Gavito M.E., Jakobsen I., Mikkelsen T.N., Mora F. (2019). Direct Evidence for Modulation of Photosynthesis by an Arbuscular Mycorrhiza-Induced Carbon Sink Strength. New Phytol..

[95] Kaschuk G., Kuyper T.W., Leffelaar P.A., Hungria M., Giller K.E. (2009). Are the Rates of Photosynthesis Stimulated by the Carbon Sink Strength of Rhizobial and Arbuscular Mycorrhizal Symbioses?. Soil Biol. Biochem..

[96] Wright D.P., Read D.J., Scholes J.D. (1998). Mycorrhizal Sink Strength Influences Whole Plant Carbon Balance of *Trifolium repens* L.. Plant Cell Environ..

[97] Hijri M. (2016). Analysis of a Large Dataset of Mycorrhiza Inoculation Field Trials on Potato Shows Highly Significant Increases in Yield. Mycorrhiza.

[98] Sabia E., Claps S., Morone G., Bruno A., Sepe L., Aleandri R. (2015). Field Inoculation of Arbuscular Mycorrhiza on Maize (*Zea mays* L.) under Low Inputs: Preliminary Study on Quantitative and Qualitative Aspects. Ital. J. Agron..

[99] Lu F.C., Lee C.Y., Wang C.L. (2015). The Influence of Arbuscularmycorrhizal Fungi Inoculation on Yam (*Dioscorea* spp.) Tuber Weights and Secondary Metabolite Content. PeerJ.

[100] Gao X., Guo H., Zhang Q., Guo H., Zhang L., Zhang C., Gou Z., Liu Y., Wei J., Chen A. (2020). Arbuscular Mycorrhizal Fungi (AMF) Enhanced the Growth, Yield, Fiber Quality and Phosphorus Regulation in Upland Cotton (*Gossypium hirsutum* L.). Sci. Rep..

[101] Zhang X., Wang L., Ma F., Yang J., Su M. (2017). Effects of Arbuscular Mycorrhizal Fungi Inoculation on Carbon and Nitrogen Distribution and Grain Yield and Nutritional Quality in Rice (*Oryza sativa* L.). J. Sci. Food Agric..

[102] Zeng L., Li J., Wang M. (2014). Effects of arbuscular mycorrhizal (AM) fungi on citrus quality under nature conditions. Southwest China J. Agric. Sci..

[103] Baslam M., Garmendia I., Goicoechea N. (2011). Arbuscular Mycorrhizal Fungi (AMF) Improved Growth and Nutritional Quality of Greenhouse-Grown Lettuce. J. Agric. Food Chem..

[104] Yuan M.L., Zhang M.H., Shi Z.Y., Yang S., Zhang M.G., Wang Z., Wu S.W., Gao J.K. (2023). Arbuscular Mycorrhizal Fungi Enhance Active Ingredients of Medicinal Plants: A Quantitative Analysis. Front. Plant Sci..

[105] Yilmaz A., Karik Ü. (2022). AMF and PGPR Enhance Yield and Secondary Metabolite Profile of Basil (*Ocimum basilicum* L.). Ind. Crops Prod..

[106] Akachoud O., Bouamama H., Facon N., Laruelle F., Zoubi B., Benkebboura A., Ghoulam C., Qaddoury A., Lounès-Hadj Sahraoui A. (2022). Mycorrhizal Inoculation Improves the Quality and Productivity of Essential Oil Distilled from Three Aromatic and Medicinal Plants: *Thymus Satureioides*, *Thymus Pallidus*, and *Lavandula Dentata*. Agronomy.

[107] Berruti A., Lumini E., Balestrini R., Bianciotto V. (2015). Arbuscular Mycorrhizal Fungi as Natural Biofertilizers: Let’s Benefit from Past Successes. Front. Microbiol..

[108] Thirkell T.J., Charters M.D., Elliott A.J., Sait S.M., Field K.J. (2017). Are Mycorrhizal Fungi Our Sustainable Saviours? Considerations for Achieving Food Security. J. Ecol..

[109] Hernández-Acosta E., Trejo-Aguilar D., Rivera-Fernández A., Ferrera-Cerrato R., Hernández-Acosta E., Trejo-Aguilar D., Rivera-Fernández A., Ferrera-Cerrato R. (2020). Arbuscular Mycorrhiza as a Biofertilizer in Production of Coffee. Terra Latinoam..

[110] Madawala H.M.S.P. (2021). Arbuscular Mycorrhizal Fungi as Biofertilizers: Current Trends, Challenges, and Future Prospects. Biofertilizers Volume 1: Advances in Bio-Inoculants.

[111] Wu S., Shi Z., Chen X., Gao J., Wang X. (2022). Arbuscular Mycorrhizal Fungi Increase Crop Yields by Improving Biomass under Rainfed Condition: A Meta-Analysis. PeerJ.

[112] Hasanuzzaman M., Gill S.S., Fujita M. (2013). Physiological Role of Nitric Oxide in Plants Grown Under Adverse Environmental Conditions. Plant Acclimation to Environmental Stress.

[113] Ahanger M.A., Tittal M., Mir R.A., Agarwal R. (2017). Alleviation of Water and Osmotic Stress-Induced Changes in Nitrogen Metabolizing Enzymes in *Triticum aestivum* L. Cultivars by Potassium. Protoplasma.

[114] Borde M., Dudhane M., Jite P.K. (2010). AM Fungi Influences the Photosynthetic Activity, Growth and Antioxidant Enzymes in *Allium sativum* L. under Salinity Condition. Not. Sci. Biol..

[115] Cheng L., Booker F.L., Tu C., Burkey K.O., Zhou L., Shew H.D., Rufty T.W., Hu S. (2012). Arbuscular Mycorrhizal Fungi Increase Organic Carbon Decomposition under Elevated CO_2_. Science.

[116] Zhu X.C., Song F.B., Liu T.D., Liu S.Q. (2010). Arbuscular Mycorrhizae Reducing Water Loss in Maize Plants under Low Temperature Stress. Plant Signal Behav..

[117] Bunn R., Lekberg Y., Zabinski C. (2009). Arbuscular Mycorrhizal Fungi Ameliorate Temperature Stress in Thermophilic Plants. Ecol..

[118] Chu X.T., Fu J.J., Sun Y.F., Xu Y.M., Miao Y.J., Xu Y.F., Hu T.M. (2016). Effect of Arbuscular Mycorrhizal Fungi Inoculation on Cold Stress-Induced Oxidative Damage in Leaves of *Elymus nutans* Griseb. S. Afr. J. Bot..

[119] Kapoor R., Sharma D., Bhatnagar A.K. (2008). Arbuscular Mycorrhizae in Micropropagation Systems and Their Potential Applications. Sci. Hortic..

[120] Evelin H., Kapoor R., Giri B. (2009). Arbuscular Mycorrhizal Fungi in Alleviation of Salt Stress: A Review. Ann. Bot..

[121] Ouziad F., Hildebrandt U., Schmelzer E., Bothe H. (2005). Differential Gene Expressions in Arbuscular Mycorrhizal-Colonized Tomato Grown under Heavy Metal Stress. J. Plant Physiol..

[122] Diagne N., Ndour M., Djighaly P.I., Ngom D., Ngom M.C.N., Ndong G., Svistoonoff S., Cherif-Silini H. (2020). Effect of Plant Growth Promoting Rhizobacteria (PGPR) and Arbuscular Mycorrhizal Fungi (AMF) on Salt Stress Tolerance of *Casuarina obesa* (Miq.). Front. Sustain. Food Syst..

[123] Wang Y., Wang M., Li Y., Wu A., Huang J. (2018). Effects of Arbuscular Mycorrhizal Fungi on Growth and Nitrogen Uptake of *Chrysanthemum morifolium* under Salt Stress. PLoS ONE.

[124] Lin A.J., Zhang X.H., Wong M.H., Ye Z.H., Lou L.Q., Wang Y.S., Zhu Y.G. (2007). Increase of Multi-Metal Tolerance of Three Leguminous Plants by Arbuscular Mycorrhizal Fungi Colonization. Env. Geochem. Health.

[125] Jones D.L., Hodge A., Kuzyakov Y. (2004). Plant and Mycorrhizal Regulation of Rhizodeposition. New Phytol..

[126] Giasson P., Karam A., Jaouich A. (2009). Arbuscular Mycorrhizae and Alleviation of Soil Stresses on Plant Growth. Mycorrhizae: Sustainable Agriculture and Forestry.

[127] Yang C., Zhao W., Wang Y., Zhang L., Huang S., Lin J. (2020). Metabolomics Analysis Reveals the Alkali Tolerance Mechanism in *Puccinellia Tenuiflora* Plants Inoculated with Arbuscular Mycorrhizal Fungi. Microorganisms.

[128] Sheng M., Tang M., Zhang F., Huang Y. (2011). Influence of Arbuscular Mycorrhiza on Organic Solutes in Maize Leaves under Salt Stress. Mycorrhiza.

[129] Al-Karaki G., McMichael B., Zak J. (2004). Field Response of Wheat to Arbuscular Mycorrhizal Fungi and Drought Stress. Mycorrhiza.

[130] Marulanda A., Azcón R., Ruiz-Lozano J.M. (2003). Contribution of Six Arbuscular Mycorrhizal Fungal Isolates to Water Uptake by *Lactuca Sativa* Plants under Drought Stress. Physiol. Plant.

[131] Neumann E., Schmid B., Römheld V., George E. (2009). Extraradical Development and Contribution to Plant Performance of an Arbuscular Mycorrhizal Symbiosis Exposed to Complete or Partial Rootzone Drying. Mycorrhiza.

[132] Maure C., Verdoucq L., Luu D.T., Santoni V. (2008). Plant Aquaporins: Membrane Channels with Multiple Integrated Functions. Annu. Rev. Plant Biol..

[133] del Mar Alguacil M., Kohler J., Caravaca F., Roldán A. (2009). Differential Effects of *Pseudomonas mendocina* and *Glomus intraradices* on Lettuce Plants Physiological Response and Aquaporin PIP2 Gene Expression under Elevated Atmospheric CO_2_ and Drought. Microb. Ecol..

[134] Liu T., Li Z., Hui C., Tang M., Zhang H. (2016). Effect of *Rhizophagus irregularis* on Osmotic Adjustment, Antioxidation and Aquaporin PIP Genes Expression of *Populus* × *Canadensis* ‘Neva’ under Drought Stress. Acta Physiol. Plant.

[135] Jia-Dong H., Tao D., Hui-Hui W., Zou Y.N., Wu Q.S., Kamil K. (2019). Mycorrhizas Induce Diverse Responses of Root TIP Aquaporin Gene Expression to Drought Stress in Trifoliate Orange. Sci. Hortic..

[136] Bárzana G., Aroca R., Bienert G.P., Chaumont F., Ruiz-Lozano J.M. (2014). New Insights into the Regulation of Aquaporins by the Arbuscular Mycorrhizal Symbiosis in Maize Plants under Drought Stress and Possible Implications for Plant Performance. Mol. Plant Microbe Interact..

[137] Quiroga G., Erice G., Aroca R., Chaumont F., Ruiz-Lozano J.M. (2017). Enhanced Drought Stress Tolerance by the Arbuscular Mycorrhizal Symbiosis in a Drought-Sensitive Maize Cultivar Is Related to a Broader and Differential Regulation of Host Plant Aquaporins than in a Drought-Tolerant Cultivar. Front. Plant Sci..

[138] Wang D., Ni Y., Xie K., Li Y., Wu W., Shan H., Cheng B., Li X. (2024). Aquaporin ZmTIP2;3 Promotes Drought Resistance of Maize through Symbiosis with Arbuscular Mycorrhizal Fungi. Int. J. Mol. Sci..

[139] Ouziad F., Wilde P., Schmelzer E., Hildebrandt U., Bothe H. (2006). Analysis of Expression of Aquaporins and Na^+^/H^+^ Transporters in Tomato Colonized by Arbuscular Mycorrhizal Fungi and Affected by Salt Stress. Environ. Exp. Bot..

[140] Liu Z., Ma L., He X., Tian C. (2014). Water Strategy of Mycorrhizal Rice at Low Temperature through the Regulation of PIP Aquaporins with the Involvement of Trehalose. Appl. Soil Ecol..

[141] Lawlor D.W., Cornic G. (2002). Photosynthetic Carbon Assimilation and Associated Metabolism in Relation to Water Deficits in Higher Plants. Plant Cell Environ..

[142] Ait-El-Mokhtar M., Laouane R.B., Anli M., Boutasknit A., Wahbi S., Meddich A. (2019). Use of Mycorrhizal Fungi in Improving Tolerance of the Date Palm (*Phoenix dactylifera* L.) Seedlings to Salt Stress. Sci. Hortic..

[143] Birhane E., Sterck F.J., Fetene M., Bongers F., Kuyper T.W. (2012). Arbuscular Mycorrhizal Fungi Enhance Photosynthesis, Water Use Efficiency, and Growth of Frankincense Seedlings under Pulsed Water Availability Conditions. Oecologia.

[144] Schubert A., Allara P., Morte A. (2004). Cleavage of Sucrose in Roots of Soybean (Glycine Max) Colonized by an Arbuscular Mycorrhizal Fungus. New Phytol..

[145] Balestrini R., Brunetti C., Chitarra W., Nerva L. (2020). Photosynthetic Traits and Nitrogen Uptake in Crops: Which Is the Role of Arbuscular Mycorrhizal Fungi?. Plants.

[146] Subramanian K.S., Charest C., Dwyer L.M., Hamilton R.I. (1997). Effects of Arbuscular Mycorrhizae on Leaf Water Potential, Sugar Content, and P Content during Drought and Recovery of Maize. Can. J. Bot..

[147] Andersen C.P., Sucoff E.I., Dixon R.K. (1987). The Influence of Low Soil Temperature on the Growth of Vesicular–Arbuscular Mycorrhizal *Fraxinuspennsylvanica*. Can. J. For. Res..

[148] Kiers E.T., Duhamel M., Beesetty Y., Mensah J.A., Franken O., Verbruggen E., Fellbaum C.R., Kowalchuk G.A., Hart M.M., Bago A. (2011). Reciprocal Rewards Stabilize Cooperation in the Mycorrhizal Symbiosis. Science.

[149] Hildebrandt U., Regvar M., Bothe H. (2007). Arbuscular Mycorrhiza and Heavy Metal Tolerance. Phytochem..

[150] Krieger-Liszkay A., Fufezan C., Trebst A. (2008). Singlet Oxygen Production in Photosystem II and Related Protection Mechanism. Photosynth. Res..

[151] Wagner D., Przybyla D., Op Den Camp R., Kim C., Landgraf F., Keun P.L., Würsch M., Laloi C., Nater M., Hideg E. (2004). The Genetic Basis of Singlet Oxygen-Induced Stress Response of *Arabidopsis thaliana*. Science.

[152] Demidchik V. (2015). Mechanisms of Oxidative Stress in Plants: From Classical Chemistry to Cell Biology. Environ. Exp. Bot..

[153] Anjum N.A., Sofo A., Scopa A., Roychoudhury A., Gill S.S., Iqbal M., Lukatkin A.S., Pereira E., Duarte A.C., Ahmad I. (2015). Lipids and Proteins—Major Targets of Oxidative Modifications in Abiotic Stressed Plants. Environ. Sci. Pollut. Res. Int..

[154] Rhoads D.M., Umbach A.L., Subbaiah C.C., Siedow J.N. (2006). Mitochondrial Reactive Oxygen Species. Contribution to Oxidative Stress and Interorganellar Signaling. Plant Physiol..

[155] Das K., Roychoudhury A. (2014). Reactive Oxygen Species (ROS) and Response of Antioxidants as ROS-Scavengers during Environmental Stress in Plants. Front. Environ. Sci..

[156] Apel K., Hirt H. (2004). Reactive Oxygen Species: Metabolism, Oxidative Stress, and Signal Transduction. Annu. Rev. Plant Biol..

[157] Rani B. (2016). Effect of Arbuscular Mycorrhiza Fungi on Biochemical Parameters in Wheat (*Triticum aestivum* L.) under Drought Conditions. Ph.D. Thesis.

[158] Abdelhameed R.E., Metwally R.A. (2019). Alleviation of Cadmium Stress by Arbuscular Mycorrhizal Symbiosis. Int. J. Phytoremediation.

[159] Li J., Meng B., Chai H., Yang X., Song W., Li S., Lu A., Zhang T., Sun W. (2019). Arbuscular Mycorrhizal Fungi Alleviate Drought Stress in C3 (*Leymus chinensis*) and C4 (*Hemarthria altissima*) Grasses via Altering Antioxidant Enzyme Activities and Photosynthesis. Front. Plant Sci..

[160] Talaat N.B., Shawky B.T. (2014). Protective Effects of Arbuscular Mycorrhizal Fungi on Wheat (*Triticum aestivum* L.) Plants Exposed to Salinity. Environ. Exp. Bot..

[161] Jahromi F., Aroca R., Porcel R., Ruiz-Lozano J.M. (2008). Influence of Salinity on the in Vitro Development of *Glomus Intraradices* and on the in Vivo Physiological and Molecular Responses of Mycorrhizal Lettuce Plants. Microb. Ecol..

[162] Kandowangko N.Y., Suryatmana G.I.A.T., Nurlaeny N., Simanungkalit R.D.M. (2009). Proline and Abscisic Acid Content in Droughted Corn Plant Inoculated with *Azospirillum* Sp. and Arbuscular Mycorrhizae Fungi. Hayati.

[163] Mohanta T.K., Bashir T., Hashem A., Abd Allah E.F. (2017). Systems Biology Approach in Plant Abiotic Stresses. Plant Physiol. Biochem..

[164] Duan X., Neuman D.S., Reiber J.M., Green C.D., Saxton A.M., Augé R.M. (1996). Mycorrhizal Influence on Hydraulic and Hormonal Factors Implicated in the Control of Stomatal Conductance during Drought. J. Exp. Bot..

[165] Hashem A., Alqarawi A.A., Radhakrishnan R., Al-Arjani A.B.F., Aldehaish H.A., Egamberdieva D., Abd Allah E.F. (2018). Arbuscular Mycorrhizal Fungi Regulate the Oxidative System, Hormones and Ionic Equilibrium to Trigger Salt Stress Tolerance in *Cucumis sativus* L.. Saudi J. Biol. Sci..

[166] Garg N., Singh S. (2018). Arbuscular Mycorrhiza *Rhizophagus irregularis* and Silicon Modulate Growth, Proline Biosynthesis and Yield in *Cajanus cajan* L. Millsp. (Pigeonpea) Genotypes Under Cadmium and Zinc Stress. J. Plant Growth Regul..

[167] Zivcak M., Brestic M., Sytar O. (2016). Osmotic Adjustment and Plant Adaptation to Drought Stress. Drought Stress Tolerance in Plants: Volume 1: Physiology Biochemistry.

[168] Chun S.C., Paramasivan M., Chandrasekaran M. (2018). Proline Accumulation Influenced by Osmotic Stress in Arbuscular Mycorrhizal Symbiotic Plants. Front. Microbiol..

[169] Ruiz-Sánchez M., Aroca R., Muñoz Y., Polón R., Ruiz-Lozano J.M. (2010). The Arbuscular Mycorrhizal Symbiosis Enhances the Photosynthetic Efficiency and the Antioxidative Response of Rice Plants Subjected to Drought Stress. J. Plant Physiol..

[170] Feng D., Wang R., Sun X., Liu L., Liu P., Tang J., Zhang C., Liu H. (2023). Heavy Metal Stress in Plants: Ways to Alleviate with Exogenous Substances. Sci. Total Environ..

[171] Gamalero E., Lingua G., Berta G., Glick B.R. (2009). Beneficial Role of Plant Growth Promoting Bacteria and Arbuscular Mycorrhizal Fungi on Plant Responses to Heavy Metal Stress. Can. J. Microbiol..

[172] Comby M., Mustafa G., Magnin-Robert M., Randoux B., Fontaine J., Reignault P., Lounès-Hadj Sahraoui A. (2017). Arbuscular Mycorrhizal Fungi as Potential Bioprotectants against Aerial Phytopathogens and Pests. Arbuscular Mycorrhizas and Stress Tolerance of Plants.

[173] Van Der Heijden M.G.A., Streitwolf-Engel R., Riedl R., Siegrist S., Neudecker A., Ineichen K., Boller T., Wiemken A., Sanders I.R. (2006). The Mycorrhizal Contribution to Plant Productivity, Plant Nutrition and Soil Structure in Experimental Grassland. New Phytol..

[174] Schouteden N., Waele D., De Panis B., Vos C.M. (2015). Arbuscular Mycorrhizal Fungi for the Biocontrol of Plant-Parasitic Nematodes: A Review of the Mechanisms Involved. Front. Microbiol..

[175] Pérez-De-Luque A., Tille S., Johnson I., Pascual-Pardo D., Ton J., Cameron D.D. (2017). The Interactive Effects of Arbuscular Mycorrhiza and Plant Growth-Promoting Rhizobacteria Synergistically Enhance Host Plant Defences against Pathogens. Sci. Rep..

[176] Lioussanne L. (2010). The Role of the Arbuscular Mycorrhiza-Associated Rhizobacteria in the Biocontrol of Soilborne Phytopathogens: A Review. SJAR.

[177] Nacoon S., Jogloy S., Riddech N., Mongkolthanaruk W., Kuyper T.W., Boonlue S. (2020). Interaction between Phosphate Solubilizing Bacteria and Arbuscular Mycorrhizal Fungi on Growth Promotion and Tuber Inulin Content of *Helianthus tuberosus* L.. Sci. Rep..

[178] Bücking H., Mensah J.A., Fellbaum C.R. (2016). Common Mycorrhizal Networks and Their Effect on the Bargaining Power of the Fungal Partner in the Arbuscular Mycorrhizal Symbiosis. Commun. Integr. Biol..

[179] Babikova Z., Gilbert L., Bruce T.J.A., Birkett M., Caulfield J.C., Woodcock C., Pickett J.A., Johnson D. (2013). Underground Signals Carried through Common Mycelial Networks Warn Neighbouring Plants of Aphid Attack. Ecol. Lett..

[180] Johnson D., Gilbert L. (2015). Interplant Signalling through Hyphal Networks. New Phytol..

[181] Song Y.Y., Zeng R.S., Xu J.F., Li J., Shen X., Yihdego W.G. (2010). Interplant Communication of Tomato Plants through Underground Common Mycorrhizal Networks. PLoS ONE.

[182] Song Y., Chen D., Lu K., Sun Z., Zeng R. (2015). Enhanced Tomato Disease Resistance Primed by Arbuscular Mycorrhizal Fungus. Front. Plant Sci..

[183] Babikova Z., Johnson D., Bruce T., Pickett J., Gilbert L. (2014). Underground Allies: How and Why Do Mycelial Networks Help Plants Defend Themselves? What Are the Fitness, Regulatory, and Practical Implications of Defence-Related Signaling between Plants via Common Mycelial Networks?. Bioessays.

[184] Zamioudis C., Pieterse C.M.J. (2012). Modulation of Host Immunity by Beneficial Microbes. Mol. Plant Microbe Interact..

[185] Zhang J., Zhou J.M. (2010). Plant Immunity Triggered by Microbial Molecular Signatures. Mol. Plant.

[186] Cameron D.D., Neal A.L., van Wees S.C.M., Ton J. (2013). Mycorrhiza-Induced Resistance: More than the Sum of Its Parts?. Trends Plant Sci..

[187] De Román M., Fernández I., Wyatt T., Sahrawy M., Heil M., Pozo M.J. (2011). Elicitation of Foliar Resistance Mechanisms Transiently Impairs Root Association with Arbuscular Mycorrhizal Fungi. J. Ecol..

[188] Ton J., Flors V., Mauch-Mani B. (2009). The Multifaceted Role of ABA in Disease Resistance. Trends Plant Sci..

[189] Trouvelot S., Bonneau L., Redecker D., van Tuinen D., Adrian M., Wipf D. (2015). Arbuscular Mycorrhiza Symbiosis in Viticulture: A Review. Agron. Sustain. Dev..

[190] Jung S.C., Martinez-Medina A., Lopez-Raez J.A., Pozo M.J. (2012). Mycorrhiza-Induced Resistance and Priming of Plant Defenses. J. Chem. Ecol..

[191] Cordier C., Gianinazzi S., Gianinazzi-Pearson V. (1996). Colonisation Patterns of Root Tissues by *Phytophthora Nicotianae* Var. Parasitica Related to Reduced Disease in Mycorrhizal Tomato. Plant Soil.

[192] Cordier C., Pozo M.J., Barea J.M., Gianinazzi S., Gianinazzi-Pearson V. (1998). Cell Defense Responses Associated with Localized and Systemic Resistance to *Phytophthora Parasitica* Induced in Tomato by an Arbuscular Mycorrhizal Fungus. MPMI.

[193] Smith S.E., Smith F.A. (2011). Roles of Arbuscular Mycorrhizas in Plant Nutrition and Growth: New Paradigms from Cellular to Ecosystem Scales. Annu. Rev. Plant Biol..

[194] Liu J., Maldonado-Mendoza I., Lopez-Meyer M., Cheung F., Town C.D., Harrison M.J. (2007). Arbuscular Mycorrhizal Symbiosis Is Accompanied by Local and Systemic Alterations in Gene Expression and an Increase in Disease Resistance in the Shoots. TPJ.

[195] Pieterse C.M.J., Leon-Reyes A., Van Der Ent S., Van Wees S.C.M. (2009). Networking by Small-Molecule Hormones in Plant Immunity. Nat. Chem. Biol..

[196] Pieterse C.M.J., Zamioudis C., Berendsen R.L., Weller D.M., Van Wees S.C.M., Bakker P.A.H.M. (2014). Induced Systemic Resistance by Beneficial Microbes. Annu. Rev. Phytopathol..

[197] Van der Ent S., Van Wees S.C.M., Pieterse C.M.J. (2009). Jasmonate Signaling in Plant Interactions with Resistance-Inducing Beneficial Microbes. Phytochemistry.

[198] Pozo M.J., Azcón-Aguilar C. (2007). Unraveling Mycorrhiza-Induced Resistance. Curr. Opin. Plant Biol..

[199] Heidel A.J., Baldwin I.T. (2004). Microarray Analysis of Salicylic Acid- and Jasmonic Acid-Signalling in Responses of *Nicotiana Attenuata* to Attack by Insects from Multiple Feeding Guilds. Plant Cell Environ..

[200] Gallou A., Lucero Mosquera H.P., Cranenbrouck S., Suárez J.P., Declerck S. (2011). Mycorrhiza Induced Resistance in Potato Plantlets Challenged by *Phytophthora Infestans*. Physiol. Mol. Plant Pathol..

[201] Wang H., Hao Z., Zhang X., Xie W., Chen B. (2022). Arbuscular Mycorrhizal Fungi Induced Plant Resistance against Fusarium Wilt in Jasmonate Biosynthesis Defective Mutant and Wild Type of Tomato. J. Fungus.

[202] Pozo M.J., Azcó n-Aguilar C., Dumas-Gaudot E., Barea J.M. (1999). B-1,3-Glucanase Activities in Tomato Roots Inoculated with Arbuscular Mycorrhizal Fungi and/or *Phytophthora Parasitica* and Their Possible Involvement in Bioprotection. Plant Sci..

[203] Jaiti F., Meddich A., El Hadrami I. (2007). Effectiveness of Arbuscular Mycorrhizal Fungi in the Protection of Date Palm (*Phoenix dactylifera* L.) against Bayoud Disease. Physiol. Mol. Plant Pathol..

[204] Fester T., Hause G. (2005). Accumulation of Reactive Oxygen Species in Arbuscular Mycorrhizal Roots. Mycorrhiza.

[205] Lee C.S., Lee Y.J., Jeun Y.C. (2005). Observations of Infection Structures on the Leaves of Cucumber Plants Pre-Treated with Arbuscular Mycorrhiza *Glomus Intraradices* after Challenge Inoculation with *Colletotrichum Orbiculare*. Plant Pathol. J..

[206] Volpin H., Elkind Y., Okon Y., Kapulnik Y. (1994). A Vesicular Arbuscular Mycorrhizal Fungus (*Glomus intraradix*) Induces a Defense Response in Alfalfa Roots. Plant Physiol..

[207] Mustafa G., Randoux B., Tisserant B., Fontaine J., Magnin-Robert M., Lounès-Hadj Sahraoui A., Reignault P. (2016). Phosphorus Supply, Arbuscular Mycorrhizal Fungal Species, and Plant Genotype Impact on the Protective Efficacy of Mycorrhizal Inoculation against Wheat Powdery Mildew. Mycorrhiza.

[208] Mora-Romero G.A., Cervantes-Gámez R.G., Galindo-Flores H., González-Ortíz M.A., Félix-Gastélum R., Maldonado-Mendoza I.E., Salinas Pérez R., León-Félix J., Martínez-Valenzuela M.C., López-Meyer M. (2015). Mycorrhiza-Induced Protection against Pathogens Is Both Genotype-Specific and Graft-Transmissible. Symbiosis.

[209] Campo S., Martín-Cardoso H., Olivé M., Pla E., Catala-Forner M., Martínez-Eixarch M., San Segundo B. (2020). Effect of Root Colonization by Arbuscular Mycorrhizal Fungi on Growth, Productivity and Blast Resistance in Rice. Rice.

[210] Wehner J., Antunes P.M., Powell J.R., Mazukatow J., Rillig M.C. (2010). Plant Pathogen Protection by Arbuscular Mycorrhizas: A Role for Fungal Diversity?. Pedobiologia.

[211] Kirk A.P., Entz M.H., Fox S.L., Tenuta M. (2011). Mycorrhizal Colonization, P Uptake and Yield of Older and Modern Wheats under Organic Management. Can. J. Plant Sci..

[212] Hetrick B.A.D., Wilson G.W.T., Cox T.S. (2011). Mycorrhizal Dependence of Modern Wheat Varieties, Landraces, and Ancestors. Canad. J. Bot..

[213] Sawers R.J.H., Gutjahr C., Paszkowski U. (2008). Cereal Mycorrhiza: An Ancient Symbiosis in Modern Agriculture. Trends Plant Sci..

[214] Parvin S., Van Geel M., Ali M.M., Yeasmin T., Lievens B., Honnay O. (2021). A Comparison of the Arbuscular Mycorrhizal Fungal Communities among Bangladeshi Modern High Yielding and Traditional Rice Varieties. Plant Soil.

[215] Blackburn T.M., Pyšek P., Bacher S., Carlton J.T., Duncan R.P., Jarošík V., Wilson J.R.U., Richardson D.M. (2011). A Proposed Unified Framework for Biological Invasions. Trends Ecol. Evol..

[216] Thomsen C., Loverock L., Kokkoris V., Holland T., Bowen P.A., Hart M. (2021). Commercial Arbuscular Mycorrhizal Fungal Inoculant Failed to Establish in a Vineyard despite Priority Advantage. PeerJ.

[217] Elliott A.J., Daniell T.J., Cameron D.D., Field K.J. (2021). A Commercial Arbuscular Mycorrhizal Inoculum Increases Root Colonization across Wheat Cultivars but Does Not Increase Assimilation of Mycorrhiza-Acquired Nutrients. Plants People Planet.

[218] Jerbi M., Labidi S., Lounes-Hadj Sahraoui A., Dalpe Y., Ben Jeddi F. (2020). Native Arbuscular Mycorrhizal Fungi Enhance Plant Growth and Productivity of Hulless Barley (*Hordeum vulgare* ssp. *Nudum* L.). J. New Sci..

[219] De Leon D.G., Vahter T., Zobel M., Koppel M., Edesi L., Davison J., Al-Quraishy S., Hozzein W.N., Moora M., Oja J. (2020). Different Wheat Cultivars Exhibit Variable Responses to Inoculation with Arbuscular Mycorrhizal Fungi from Organic and Conventional Farms. PLoS ONE.

[220] Séry D.J.M., Kouadjo Z.G.C., Voko B.R.R., Zézé A. (2016). Selecting Native Arbuscular Mycorrhizal Fungi to Promote Cassava Growth and Increase Yield under Field Conditions. Front. Microbiol..

[221] Frew A. (2021). Contrasting Effects of Commercial and Native Arbuscular Mycorrhizal Fungal Inoculants on Plant Biomass Allocation, Nutrients, and Phenolics. Plants People Planet.

[222] Ijdo M., Schtickzelle N., Cranenbrouck S., Declerck S. (2010). Do Arbuscular Mycorrhizal Fungi with Contrasting Life-History Strategies Differ in Their Responses to Repeated Defoliation?. FEMS Microbiol. Ecol..

[223] Begon M., Harper J.L., Townsend C.R. (1996). Ecology: Individuals, Populations, and Communities.

[224] Hart M.M., Reader R.J., Klironomos J.N. (2001). Life-History Strategies of Arbuscular Mycorrhizal Fungi in Relation to Their Successional Dynamics. Mycologia.

[225] Klironomos J.N., Hart M.M. (2002). Colonization of Roots by Arbuscular Mycorrhizal Fungi Using Different Sources of Inoculum. Mycorrhiza.

[226] de Souza F.A., Dalpé Y., Declerck S., de la Providencia I.E., Séjalon-Delmas N. (2005). Life History Strategies in *Gigasporaceae*: Insight from Monoxenic Culture. In Vitro Culture of Mycorrhizas.

[227] Kinnunen M., Dechesne A., Proctor C., Hammes F., Johnson D., Quintela-Baluja M., Graham D., Daffonchio D., Fodelianakis S., Hahn N. (2016). A Conceptual Framework for Invasion in Microbial Communities. ISME J..

[228] Declerck S., D’or D., Cranenbrouck S., Boulengé L.E. (2001). Modelling the Sporulation Dynamics of Arbuscular Mycorrhizal Fungi in Monoxenic Culture. Mycorrhiza.

[229] Hart M.M., Reader R.J. (2002). Taxonomic Basis for Variation in the Colonization Strategy of Arbuscular Mycorrhizal Fungi. New Phytol..

[230] Maherali H., Klironomos J.N. (2007). Influence of Phylogeny on Fungal Community Assembly and Ecosystem Functioning. Science.

[231] van der Heyde M., Ohsowski B., Abbott L.K., Hart M. (2017). Arbuscular Mycorrhizal Fungus Responses to Disturbance Are Context-Dependent. Mycorrhiza.

[232] Basiru S., Hijri M. (2022). Does Commercial Inoculation Promote Arbuscular Mycorrhizal Fungi Invasion?. Microorganisms.

[233] Middleton E.L., Richardson S., Koziol L., Palmer C.E., Yermakov Z., Henning J.A., Schultz P.A., Bever J.D., Middleton E.L., Richardson S. (2015). Locally Adapted Arbuscular Mycorrhizal Fungi Improve Vigor and Resistance to Herbivory of Native Prairie Plant Species. Ecosphere.

[234] Lutz S., Bodenhausen N., Hess J., Valzano-Held A., Waelchli J., Deslandes-Hérold G., Schlaeppi K., van der Heijden M.G.A. (2023). Soil Microbiome Indicators Can Predict Crop Growth Response to Large-Scale Inoculation with Arbuscular Mycorrhizal Fungi. Nat. Microbiol..

[235] Campagnac E., Fontaine J., Sahraoui A.L.H., Laruelle F., Durand R., Grandmougin-Ferjani A. (2008). Differential Effects of Fenpropimorph and Fenhexamid, Two Sterol Biosynthesis Inhibitor Fungicides, on Arbuscular Mycorrhizal Development and Sterol Metabolism in Carrot Roots. Phytochemistry.

[236] Calonne M., Sahraoui A.L.H., Campagnac E., Debiane D., Laruelle F., Grandmougin-Ferjani A., Fontaine J. (2012). Propiconazole Inhibits the Sterol 14α-Demethylase in Glomus Irregulare like in Phytopathogenic Fungi. Chemosphere.

[237] Olsson P.A., Bååth E., Jakobsen I. (1997). Phosphorus Effects on the Mycelium and Storage Structures of an Arbuscular Mycorrhizal Fungus as Studied in the Soil and Roots by Analysis of Fatty Acid Signatures. Appl. Environ. Microbiol..

[238] Le Tacon F., Le Tacon T., Mauron V., Rousseau Y., Backer M., Bouchard D. Fertilisation Raisonnée et Mycorhize. Proceedings of the 4ème Rencontre de la Fertilisation Raisonnée.

[239] Lin X., Feng Y., Zhang H., Chen R., Wang J., Zhang J., Chu H. (2012). Long-Term Balanced Fertilization Decreases Arbuscular Mycorrhizal Fungal Diversity in an Arable Soil in North China Revealed by 454 Pyrosequencing. Environ. Sci. Technol..

[240] Verbruggen E., van der Heijden M.G.A., Rillig M.C., Kiers E.T. (2013). Mycorrhizal Fungal Establishment in Agricultural Soils: Factors Determining Inoculation Success. New Phytol..

[241] Fritz M., Jakobsen I., Lyngkjær M.F., Thordal-Christensen H., Pons-Kühnemann J. (2006). Arbuscular Mycorrhiza Reduces Susceptibility of Tomato to *Alternaria Solani*. Mycorrhiza.

[242] Douds D.D., Galvez L., Janke R.R., Wagoner P. (1995). Effect of Tillage and Farming System upon Populations and Distribution of Vesicular-Arbuscular Mycorrhizal Fungi. Agric. Ecosyst. Environ..

[243] Jansa J., Mozafar A., Anken T., Ruh R., Sanders I.R., Frossard E. (2002). Diversity and Structure of AMF Communities as Affected by Tillage in a Temperate Soil. Mycorrhiza.

[244] Jansa J., Mozafar A., Kuhn G., Anken T., Ruh R., Sanders I.R., Frossard E. (2003). Soil Tillage Affects the Community of Mycorrhizal Fungi in Maize Roots. Ecol. Appl..

[245] Oehl F., Sieverding E., Ineichen K., Mäder P., Boller T., Wiemken A. (2003). Impact of Land Use Intensity on the Species Diversity of Arbuscular Mycorrhizal Fungi in Agroecosystems of Central Europe. Appl. Environ. Microbiol..

[246] Torres-Arias Y., Fors R.O., Nobre C., Gómez E.F., Berbara R.L.L. (2017). Production of Native Arbuscular Mycorrhizal Fungi Inoculum under Different Environmental Conditions. Braz. J. Microbiol..

[247] Mycorrhiza-based Biofertilizer Market Growth Trends and Forecast (2020–2025) ReportLinker Organic Fertilizer Industry 2024. https://www.reportlinker.com/market-report/Fertilizer/87464/Organic-Fertilizer.

[248] Basiru S., Mwanza H.P., Hijri M. (2020). Analysis of Arbuscular Mycorrhizal Fungal Inoculant Benchmarks. Microorganisms.

[249] INRAE (2017). Fiche Technique (2): Multiplier Des Champignons Mycorhiziens Sur Son Exploitation.

[250] Tawaraya K., Hirose R., Wagatsuma T. (2012). Inoculation of Arbuscular Mycorrhizal Fungi Can Substantially Reduce Phosphate Fertilizer Application to *Allium Fistulosum* L. and Achieve Marketable Yield under Field Condition. Biol. Fertil. Soils.

[251] Maiti D., Singh R.K., Variar M. (2012). Rice-Based Crop Rotation for Enhancing Native Arbuscular Mycorrhizal (AM) Activity to Improve Phosphorus Nutrition of Upland Rice (*Oryza sativa* L.). Biol. Fertil. Soils.

[252] Jones F.R. (1924). A Mycorrhizal Fungus in the Roots of Legumes and Some Other Plants. J. Agric. Res..

[253] Rayner M.C. (1926). Mycorrhiza. New Phytol..

[254] Harley J.L. (1991). The History of Research on Mycorrhiza and the Part Played by Professor Beniamino Peyronel. Estratto da Funghi, Piante e Suolo, Quarat’anni di Ricerche del centro di Studio sulla Micologia del Terreno nel Centenario della Nascita del suo Fondatore Beniamino Peyronel.

[255] Mosse B., Hepper C. (1975). Vesicular-Arbuscular Mycorrhizal Infections in Root Organ Cultures. Physiol. Plant Pathol..

[256] Sharma S., Sharma S., Aggarwal A., Sharma V., Singh M., Kaushik S. (2017). Mass Multiplication of Arbuscular Mycorrhizal Fungi. Mycorrhizal Fungi.

[257] Kučová L., Záhora J., Pokluda R. (2016). Effect of Mycorrhizal Inoculation of Leek *Allium Porrum* L. on Mineral Nitrogen Leaching. Hortic. Sci..

[258] Selvakumar G., Kim K., Walitang D., Chanratana M., Kang Y., Chung B., Sa T. (2016). Trap Culture Technique for Propagation of Arbuscular Mycorrhizal Fungi Using Different Host Plants. KJSSF.

[259] Sayeed Akhtar M., Nor S., Abdullah A. (2014). Mass Production Techniques of Arbuscular Mycorrhizal Fungi: Major Advantages and Disadvantages: A Review. Biosci. Biotechnol. Res. Asia.

[260] Kumar A., Singh R., Adholeya A. (2017). Biotechnological Advancements in Industrial Production of Arbuscular Mycorrhizal Fungi: Achievements, Challenges, and Future Prospects. Developments in Fungal Biology and Applied Mycology.

[261] Morrison S., Walker B.K. (1990). Production of Mycorrhizal Inoculum by Static Culture Hydroponics.

[262] Lee Y.J., George E. (2005). Development of a Nutrient Film Technique Culture System for Arbuscular Mycorrhizal Plants. HortScience.

[263] Mosse B., Thompson J.P. (1980). Production of Mycorrhizal Fungi. J. Gen. Microbiol..

[264] Sylvia D.M., Hubbell D.H. (1986). Growth and Sporulation of Vesicular-Arbuscular Mycorrhizal Fungi in Aeroponic and Membrane Systems. Symbiosis.

[265] Jarstfer A.G., Sylvia D.M. (1999). Aeroponic Culture of VAM Fungi. Mycorrhiza.

[266] Bécard G., Fortin J.A. (1988). Early Events of Vesicular-Arbuscular Mycorrhiza Formation on Ri T-DNA Transformed Roots. New Phytol..

[267] Wang W.-K. (2004). Method of Facilitating Mass Production and Sporulation of Arbuscular Mycorrhizal Fungi Aseptic in Vitro. U.S. Patent.

[268] Jolicoeur M., Williams R.D., Chavarie C., Fortin J.A., Archambault J. (1999). Production of Glomus Intraradices Propagules, an Arbuscular Mycorrhizal Fungus, in an Airlift Bioreactor. Biotechnol. Bioeng..

[269] Fortin J.A., Declerck S., Strullu D.-G. (2005). In Vitro Culture of Mycorrhizas. In Vitro Culture of Mycorrhizas.

[270] Paré L., Banchini C., Hamel C., Bernier L., Stefani F. (2022). A Simple and Low-Cost Technique to Initiate Single-Spore Cultures of Arbuscular Mycorrhizal Fungi Using a Superabsorbent Polymer. Symbiosis.

[271] Voets L., De Boulois H.D., Renard L., Strullu D.G., Declerck S. (2005). Development of an Autotrophic Culture System for the in Vitro Mycorrhization of Potato Plantlets. FEMS Microbiol. Lett..

[272] Gargouri M., Bates P.D., Declerck S. (2021). Combinatorial Reprogramming of Lipid Metabolism in Plants: A Way towards Mass-production of Bio-fortified Arbuscular Mycorrhizal Fungi Inoculants. Microb. Biotechnol..

[273] Hooker J.E., Jaizme-Vega M., Atkinson D. (1994). Biocontrol of Plant Pathogens Using Arbuscular Mycorrhizal Fungi. Impact of Arbuscular Mycorrhizas on Sustainable Agriculture and Natural Ecosystems.

[274] Oviatt P., Rillig M.C. (2021). Mycorrhizal Technologies for an Agriculture of the Middle. Plants People Planet.

[275] Bender S.F., Wagg C., van der Heijden M.G.A. (2016). An Underground Revolution: Biodiversity and Soil Ecological Engineering for Agricultural Sustainability. Trends Ecol. Evol..

[276] Lekberg Y., Helgason T. (2018). In Situ Mycorrhizal Function—Knowledge Gaps and Future Directions. New Phytol..

